# Effect of the surface morphology of alkaline-earth metal oxides on the oxidative coupling of methane

**DOI:** 10.1080/14686996.2024.2435801

**Published:** 2024-12-20

**Authors:** Nobutsugu Hamamoto, Takakazu Kawahara, Ryoto Hagiwara, Kohei Matsuo, Kodai Matsukawa, Yoyo Hinuma, Takashi Toyao, Ken-Ichi Shimizu, Takashi Kamachi

**Affiliations:** a Department of Applied Chemistry, Faculty of Engineering, Sanyo-Onoda City University, Sanyo-Onoda, Japan; b Department of Life, Environment and Applied Chemistry, Fukuoka Institute of Technology, Fukuoka, Japan; c Department of Energy and Environment, National Institute of Advanced Industrial Science and Technology (AIST), Ikeda, Osaka, Japan; d Institute for Catalysis, Hokkaido University, Sapporo, Hokkaido, Japan

**Keywords:** First-principles calculation, alkaline-earth metal oxide, step surface, octopolar surface

## Abstract

Alkaline-earth metal oxides with the rocksalt structure, which are simple ionic solids, have attracted attention in attempts to gain fundamental insights into the properties of metal oxides. The surfaces of alkaline-earth metal oxides are considered promising catalysts for the oxidative coupling of methane (OCM); however, the development of such catalysts remains a central research topic. In this paper, we performed first-principles calculations to investigate the ability of four alkaline-earth metal oxides (MgO, CaO, SrO, and BaO) to catalyze the OCM. We adopted five types of surfaces of rocksalt phases as research targets: the (100), (110), stepped (100), oxygen-terminated octopolar (111), and metal-terminated octopolar (111) surfaces. We found that the formation energy of surface O vacancies is a good descriptor for the adsorption energy of a H atom and a methyl radical. The energies related to the OCM mechanism show that, compared with the most stable surface, the minor surfaces better promote the C – H bond cleavage of methane. However, as the trade-off for this advantage, the minor surfaces exhibit increased affinity for the methyl radical. On the basis of this trade-off relationship between properties, we identified several surfaces that we expect to be promising OCM catalysts. Our investigation of the temperature dependence of the Gibbs free energy indicated that, at higher temperatures, the step (100) surface exhibits properties that might benefit the OCM mechanism.

## IMPACT STATEMENT

This theoretical study employs DFT calculations and data-driven insights to identify promising alkaline-earth metal oxide surfaces for OCM catalysts, highlighting surface O vacancy formation energies and entropic effects.

## GRAPHICAL ABSTRACT

**Figure:**
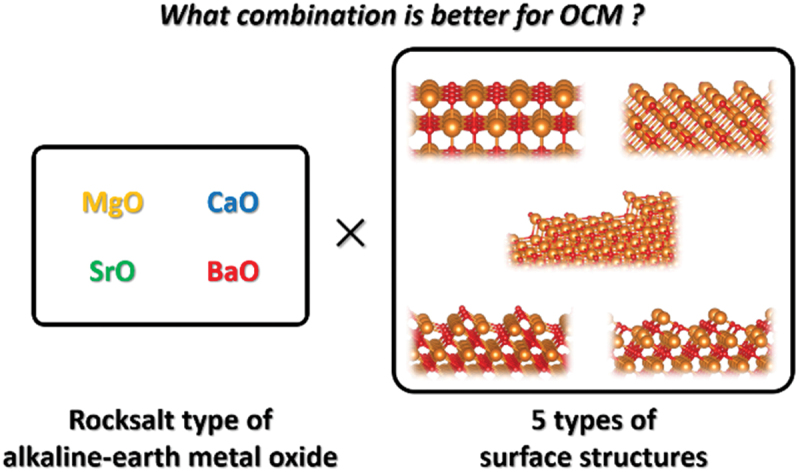

## 1. Introduction

The oxidative coupling of methane (OCM) is a chemical reaction used to produce value-added chemicals such as ethane (C_2_H_6_) from methane (CH_4_); however, numerous studies have shown that this reaction does not proceed easily. One reason for this difficulty is the high strength of the *sp*^3^ C – H bond of CH_4_; another reason is that controlling this process is difficult. The nonselective oxidation of CH_4_ produces undesired compounds such as CO_*x*_. The development of OCM catalysts with high efficiency and selectivity remains a challenge in the chemical industry.

Metal oxides have attracted intensive attention because of their wide range of technological applications, including catalysis of the OCM, which is a consequence of their surface exhibiting two active sites: Lewis acid and Lewis base sites. Previous studies have indicated that effective catalysts for the OCM should exhibit good reactivity and high selectivity. In this regard, the difference in the behavior of CH_4_ on a PdO surface and that of CH_4_ on a MgO surface has often been compared. The PdO surface is known to be active toward the C – H bond cleavage of alkanes [1–4]. However, the activity of the PdO surface toward the OCM reaction and the controllability of the oxidation of CH_4_ via the OCM reaction exhibit a trade-off relationship. Although the MgO surface is known to exhibit good selectivity for the OCM reaction [5], high temperatures are required for this surface to activate the C – H bonds of CH_4_; that is, the MgO surface exhibits poor activity. Because activity and selectivity fundamentally exhibit a trade-off relationship, the search continues for innovative catalysts that overcome this problem.

Numerous studies have been conducted to find a novel surface with high catalytic activity. Examples of the investigated approaches include doping of metal cations [6–9] or inorganic elements [10–13], using metal oxides as support materials [14–21], preparing metal – insulator – metal (MIM) systems [22–26], preparing nanoparticles [27–29], and using softer oxidants instead of oxygen [30]. In addition to these approaches, activated surfaces with low-coordinated atoms have been intensively investigated. For example, we previously reported that a TiO_2_ surface with oxygen defects, which involves unfavorable coordination environments, is expected to exhibit high catalytic activity [31,32]. The high-index surfaces also show strong potential as catalysts with substantial activity [33]. The origin of the activity that the minor surfaces exhibit must be understood to advance the development of novel catalysts.

**Figure 1.**
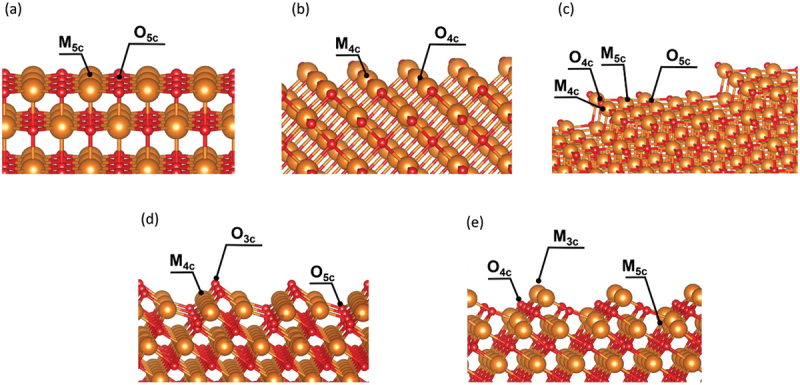
The surface structures focused on in the present paper: (a) the (100) surface, (b) (110) surface, (c) stepped (100) surface, (d) O-oct surface, and (e) M-oct surface. The orange and red spheres indicate alkaline-earth metal and oxygen atoms, respectively.

Alkaline-earth metal oxides with the rocksalt structure, which are simple ionic solids, have attracted much attention in both experimental and theoretical studies designed to obtain fundamental information about various metal oxides [34,35]. Although these oxides are expected to serve as catalysts for the OCM, this application is limited to high temperatures because of these oxides’ high stability. Improving the catalytic activity of alkaline-earth metal oxides for the OCM remains a central research topic. The importance of the morphology of OCM catalysts has been highlighted [36]. We here note five types of surface structures of rocksalt phases (Figure 1) and briefly describe the related works published thus far.

The (100) surface is known to be the most stable rocksalt surface because of its close-packed structure that resembles the corresponding bulk [37]. This surface has been vigorously investigated because of its high stability and ease of experimental preparation [38,39]. Understanding the OCM reaction mechanism on this surface would provide basic information about the mechanisms on other surfaces. However, actual surfaces do not necessarily consist of only the most stable surface. The existence of defects such as vacancies, steps, and adatoms on a real surface must be considered. Stepped high-index surfaces of alkaline-earth metal oxides have been identified by experimental methods, including transmission electron microscopy (TEM) and atomic force microscopy (AFM) [40–45]. Defect surfaces that include step sites are known to play an important role in molecular adsorption and catalyst function [46]. For example, Leeuw et al. have reported that the dissociative adsorption of H_2_O preferentially occurs at the edge of the step surface of MgO and CaO [47]. Schwach et al. showed that sites that strongly adsorb CH_4_ exist on the MgO surfaces of higher-index planes and proposed a mechanism of CH_4_ activation on the MgO step surface [48]. From these perspectives, an investigation of the character of atoms on such step surfaces is critical to understanding phenomena that occur on the actual surfaces.

The (100) surface is a type I nonpolar surface in Tasker’s classification [37]; the (110) surface is also a type I nonpolar surface but has a higher surface energy than the (100) surface. The (110) surface often attracts attention in the case of Li-doped alkaline-earth metal oxide surfaces, which are well known to be effective catalysts for the OCM [49–51]. Li-doped MgO catalysts have been speculated to exhibit high activity for the OCM because of their [Li^+^O^−^] sites [51]. The breaking of the *sp*^3^ C – H bonds on various Li-doped MgO surface models, including models with a Li atom in the outermost surface or second layer on MgO(100), (110), or (111) surfaces, near an oxygen vacancy, or on a step-edge part, has been theoretically investigated [52–60]. However, the addition of Li into MgO has been reported to alter the microstructures of the MgO surface, improving its catalytic activity toward the OCM [61–66]. These reports show that the Li additive increases the surface density of the fourfold-coordinated Mg site of MgO {110} facets and that this site is responsible for the high activity and selectivity in the OCM reaction. Compared with the (100) surface, the (110) surface is expected to exhibit greater activity for molecules such as CH_4_, as evidenced by the energies associated with the molecular or dissociative adsorption of CO, CO_2_, and H_2_O [67–70]. Further investigation of the (110) surface, which is less stable than the most stable surface, (100), and is an experimentally realizable surface, is expected to provide helpful information for developing novel catalysts with high catalytic activity.

The bulk-truncated (111) surface, which is a type III polar surface in Tasker’s classification [37] and is considered to be unstable because of its polarity, is known to cause structural reconstruction. For example, Plass et al. experimentally reported the air-stable reconstruction surface of the MgO (111) surface via transmission high-energy electron diffraction: ($\sqrt{3}\times \sqrt{3}$)R30°, ($2\sqrt{3}\times 2\sqrt{3}$)R30°, and (2×2) [71]. Wolf theoretically proposed a (2×2) reconstruction surface, which is known as an octopolar surface, as a stable surface configuration [72,73] in which three-fourths of the top layer and one-fourth of the second layer are missing. Two models have been proposed for the octopolar surface: metal-terminated and oxygen-terminated surfaces (referred to as M-oct and O-oct, respectively). Finocchi et al. showed that an O-oct surface of MgO coexists with the M-oct α phase [74]. Ciston et al. revealed the presence of the O-oct surface of MgO under nonaqueous conditions [75], and Zhang et al. reported that the O-oct surface of MgO is the favored surface under O-rich conditions [76]. In these reports, the octopolar (111) surface of MgO was found to prefer the O-oct surface to the M-oct surface. In addition, Bajdich et al. reported in a theoretical study that the other alkaline-earth metal oxides exhibit the opposite tendency as MgO: the surface energy of the M-oct surface is lower than that of the O-oct surface [34]. Although the properties of the octopolar (111) surface for several metal oxides (including MgO) have been reported both experimentally and theoretically, as far as we know, a systematic investigation of the OCM on these surfaces has not yet been reported. Because the octopolar (111) surface has a special atom, it is expected to show catalytic properties that the other surfaces under consideration in this report lack. A detailed investigation of the characteristics of this surface should therefore provide new insights.

The C – H bond activation in the OCM proceeds via the formation of a methyl radical, which can couple to form C_2_H_6_ in the gas phase. The OCM mechanism is assumed to be given in the following steps:

$$CH_{4}+{\;}^{*}\;\to \;CH_{3}+\;H^{*}$$

$$2CH_{3}\to \;C_{2}H_{6}$$

$$2H^{*}\;\to \;H_{2}O\;+{\;}^{*}v$$

$$\frac{1}{2}O_{2}+{\;}^{*}v\to^{*}$$

where * represents the catalyst surface and *v indicates the catalyst surface with an O vacancy. We assume that C_2_H_6_ is formed through methyl radical recombination in the gas phase. Among these steps, the initial C – H bond activation one has been shown to be rate limiting for OCM [77].

In the present study, we use first-principles calculations to investigate the dependence of catalysis of the OCM reaction on four alkaline-earth metal oxides – MgO, CaO, SrO, and BaO – on the five types of surface structures of the rocksalt phase (i.e. the (100), (110), stepped (100), O-oct, and M-oct surfaces). To assess the catalytic activity of these surfaces toward the OCM reaction, we explore the most stable configuration of two adsorbates (a methyl radical and H atom), which are produced by the cleavage of the C – H bond of CH_4_, on these surfaces. There are two main reasons why we especially pay attention to adsorption of these species: the first reason is that the bonding strength between these species and metal oxide surfaces is associated with the catalyst poisoning. Another reason is that the cleavage of the *sp*^3^ C – H bond of CH_4_ is rate determining step to produce higher value chemicals aforementioned above, and its energy can be efficiently estimated by using the hydrogen binding energy on the basis of the Brønsted – Evans – Polanyi relationship. The importance of adsorption of these species can be also mentioned in other reports [78–81]. In addition, we use statistical analysis to examine the obtained results. Finally, the temperature effect on the OCM reaction is theoretically assessed on the basis of the Gibbs free energies.

## 2. Computational method

First-principles calculations were performed using the projector-augmented wave (PAW) method [82], as implemented in the Vienna Ab initio Simulation Package (VASP) [83,84]. We adopted the generalized gradient approximation (GGA) with the PBEsol functional, which provides a reasonable approximation of crystal structures, as shown in our previous work on binary oxides [85], as the electronic exchange – correlation functional. In addition, the empirical Grimme D3 method [86] with Becke – Johnson damping [87] was used to estimate the van der Waals interaction between the metal oxide surface and the adsorbed molecules. The Brillouin zone was sampled with 2π × 0.03 Å^−1^ in the Monkhorst – Pack scheme. A 12 Å-thick vacuum layer in the *Z*-direction was applied to avoid interaction with the image atoms. The slab models used in the present paper were obtained using the algorithm of Hinuma *et al* [88]. The step models were prepared using the automatic generation method that we recently developed [89].

The surface energy (*E*_surf_) is defined as

$$E_{\mathrm{surf}}=\frac{\left(E_{\mathrm{slab}}-E_{\mathrm{bulk}}\right)}{2A}$$

where *E*_slab_ and *E*_bulk_ are the energy of the slab and the energy of the constituents of the slab, respectively, and *A* is the in-plane area of the slab (the coefficient of 2 accounts for both sides of the slab).

The O-vacancy formation energy (*E*_Ovac_) is defined as

$$E_{\mathrm{Ovac}}=\frac{E_{\mathrm{removed}}-E_{\mathrm{slab}}+2\mu_{O}}{2}$$

where *E*_slab_, *E*_removed_, and *μ*_O_ are the energy of the slab without an O vacancy, the energy of the slab with O vacancies when two O atoms are removed (one O from each surface), and the chemical potential of the O that is removed, respectively. The removed atom is O, and the chemical potential is referenced to O_2_ gas in the current case.

The adsorption energy (*E*_ads_) of the adsorbates is defined as

$$E_{\mathrm{ads}}=\frac{E_{M/S}-2E_{M}+E{\;}_{S}}{2}$$

where M and S represents an adsorbate and the surface, respectively. The coefficient of 2 accounts for both sides of the slab.

To study the distribution of unpair electrons on surface slabs with O_vac_, adsorbed CH_3_, and adsorbed H atoms, we calculated spin densities for these models, defined as the difference between up-spin density and down-spin densities. The yellow (blue) color represented an excess of up-(down-)electron density.

A hydrogen abstraction from a CH_4_ molecule to a metal oxide surface is considered to be a key step in the OCM as shown above. To estimate the C – H activation energy of CH_4_ (*E*_act_), we used the ‘pseudo-steady-state approach’ in conjunction with the Brønsted – Evans – Polanyi relationship [90]:

$$E_{\mathrm{act}}=\frac{(E_{2H^{*}}+2E_{CH_{3}})-(E{\;}_{S}+2E_{CH_{4}})}{2}$$

where E_2H_ is the energy of a surface slab with adsorbed H atoms, E_s_ is the energy of the bare surface slab, and E_CH_3__ and E_CH_4__ are the energies of an isolated methyl radical and a CH_4_ molecule, respectively. Kumar et al. have shown that the barrier for methane activation for metal oxides with or without doping exhibit a Brønsted – Evans – Polanyi relationship with the *E*_act_ value and demonstrated that this correlation exists across a wide range of metal oxides [91,92]. This approach is expected to be a useful method to evaluate the *E*_act_ value efficiently. On the basis of these previous reports, we adopt the pseudo-steady-state approach in the present study to estimate the activation barrier of C – H breaking. Because we adopted a mirrored slab consisting of the surface sandwiched by the adsorbates, which is a reasonable model for simulating the real system, obtaining transition-state structures for this model to accurately evaluate the activation barrier of C – H breaking is difficult. We therefore adopted the pseudo-steady-state approach.

The Gibbs free energy of each surface model and molecule was calculated using vibrational frequency analysis on the basis of harmonic normal mode approximation by the finite difference method. For a surface with or without adsorbates, among the calculated 3*N* pieces of modes, we assumed that there exist 3*N* − 3 vibrational modes and the other three modes. When calculating the Gibbs free energy of the periodic surface models, we used the 3*N* − 3 vibrational modes to calculate the vibrational contributions to the entropy. The H atom and the methyl radical in the gas phase were calculated using the ideal gas approximation at several temperatures.

## 3. Results and discussion

### 3.1. Surface structure

In the first part of this work, we browse the surface structures under consideration (Figure 1). The (100) surface, which is well known as the most stable surface (Figure 1(a)), consists of fivefold-coordinated alkaline-earth metal atoms (referred to as **M**_**5c**_) and fivefold-coordinated O atoms (referred to as **O**_**5c**_). Compared with the most stable surface, other surfaces have more low-coordinated atoms. On the (110) surface (Figure 1(b)), there are fourfold-coordinated alkaline-earth metal atoms (referred to as **M**_**4c**_) and fourfold-coordinated O atoms (referred to as **O**_**4c**_). The stepped (100) surface (Figure 1(c)), which is modeled by MgO (510), has the **M**_**4c**_ and **O**_**4c**_ on the step-edge part as low-coordinated atoms, which is the same coordination environment as that on the (110) surface. **M**_**5c**_ and **O**_**5c**_ are also present on the terrace in this model because this step can be regarded as a defect on the (100) surface. The steps are 10.7, 12.2, 13.1, and 14.1 Å apart in the MgO, CaO, SrO, and BaO models, respectively, which are sufficient distances for the interaction between steps to be ignored. In the case of the octopolar (111) surface, two different surface types are known: the O-oct surface and the M-oct surface. The model of the O-oct surface (Figure 1(d)) includes threefold-coordinated O atoms (referred to as **O**_**3c**_), which are the O atoms with the smallest coordination number among the surface O atoms considered in the present work. The **O**_**5c**_ atom, whose coordination environment is similar to that on the (100) surface, also exists. Regarding the metal atom (**M**_**4c**_) on this surface, its coordination environment is identical to that on the (110) surface. The M-oct surface (Figure 1(e)), which is analogous to the oxygen-terminated one, contains threefold-coordinated alkaline-earth metal atoms (referred to as **M**_**3c**_), **M**_**5c**_, and **O**_**4c**_ atoms. We evaluated the stabilization of the two types of (111) surface models using the *E*_surf_ value; the results are compiled in Table 1. Although MgO prefers the O-oct surface to the M-oct one, the stability between these surfaces is reversed for the other alkaline-earth metal oxides. This result is in agreement with the results of Bajdich et al. [34].

**Table 1.** *E*_surf_ values of the oxygen- or metal-terminated octopolar (111) surfaces.

	Oxygen terminated	Metal terminated
MgO	140	148
CaO	97	94
SrO	76	71
BaO	47	41
		Unit: meV/Å^2^

### 3.2. O-Vacancy formation energy

**Figure 2.**
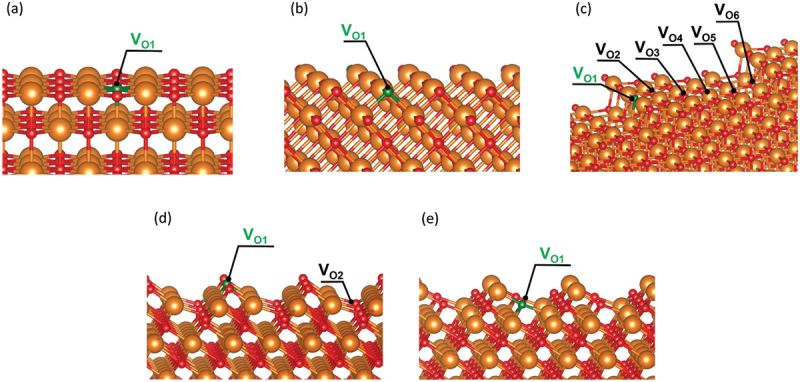
Labels of oxygen atoms on surface: (a) (100) surface, (b) (110) surface, (c) stepped (100) surface, (d) O-oct surface, and (e) M-oct surface. The orange and red spheres indicate alkaline-earth metal and oxygen atoms, respectively. The oxygen atom with the minimum *E*_Ovac_ value is green.

**Figure 3.**
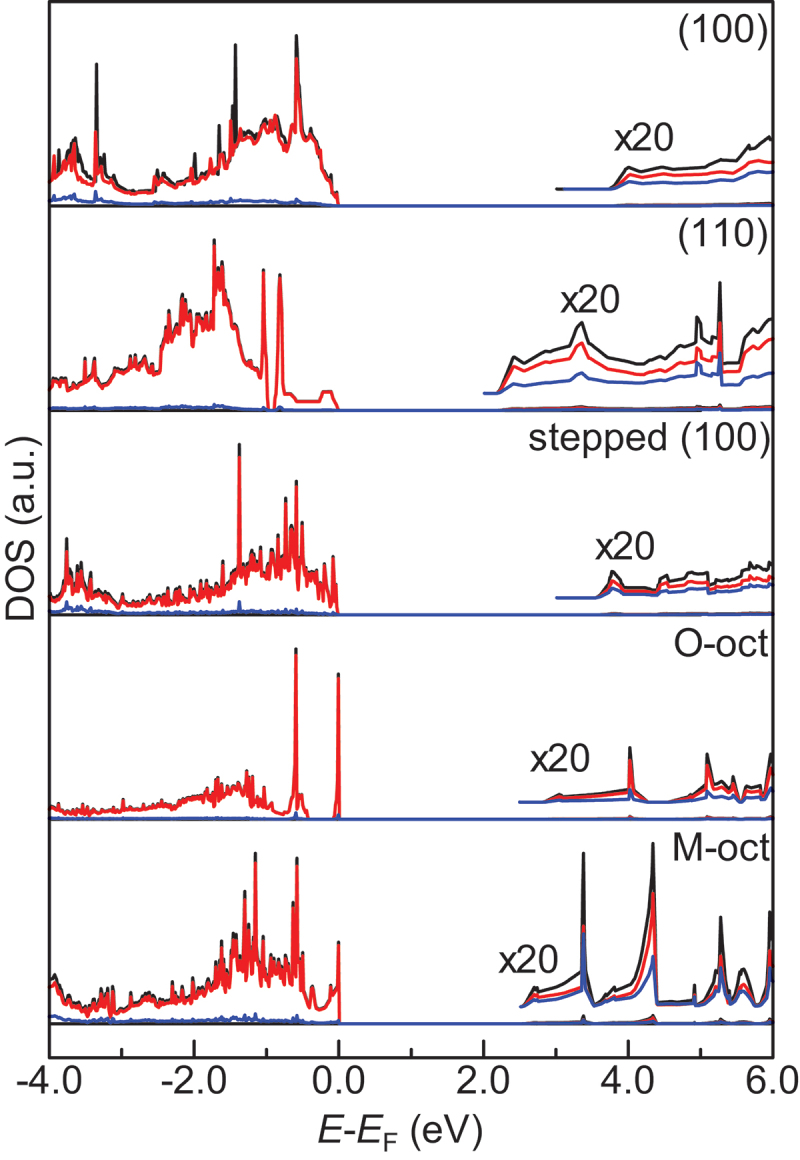
Total and partial DOS plots for the MgO surfaces under consideration in the present study. The black lines show the total DOS, and red and blue lines represent the partial DOS of the O and Mg atoms, respectively. The DOS plots multiplied by 20 are also depicted for the neighborhood of the CBM. The energy values were determined from the Fermi level.

Thus far, there are many reports to investigate the behavior of a single O-vacancy [93–102]. For example, it was reported that color centers in the near-surface region are involved in the key step of the OCM reaction [103,104]. The formation of surface OH groups is reported to facilitate the creation of an O-vacancy [105,106]. In addition, several researches have tried to investigate the double O-vacancies both in the bulk and on the surface [107,108]. However, scope of experimental observation of O-vacancies have been limited yet because of the necessity of sophisticated techniques. The *E*_Ovac_ value, which is defined as the energy required to form a single O-vacancy in this paper, often serves as a computational measurement of the reducibility of a metal oxide surface. From this perspective, this parameter has been often utilized to evaluate catalytic activity of different kinds of metal oxide surfaces. We also estimated the *E*_Ovac_ values for the various surfaces considered in the present work (Table 2) in order to investigate the difference in catalytic performance. The values for a perfect surface were taken from our previous study [93]. Among various physicochemical properties, the Kohn – Sham bandgap (BG) is most useful as a descriptor for predicting *E*_Ovac_ using machine learning [93,109,110]. Figure S1 illustrates the correlation between the *E*_Ovac_ and BG on the basis of a simple linear regression analysis. The labels of the O-vacancy positions are depicted in Figure 2, where the O atom with the minimum *E*_Ovac_ value is green. Figure S2 depicts the density of states (DOS) of the five types of surface structures under consideration for all of the alkaline-earth metal oxides. Figure 3 shows the DOS of MgO as an example.

**Table 2.** *E*_Ovac_ values for various surfaces.

		MgO	CaO	SrO	BaO
(100)	V_O1_	6.27	6.17	5.60	5.09
(110)	V_O1_	4.97	5.19	4.48	4.05
stepped (100)	V_O1_	5.48	5.33	4.75	4.31
	V_O2_	6.27	6.23	5.66	5.06
	V_O3_	6.35	6.17	5.61	4.91
	V_O4_	6.34	6.22	5.67	5.01
	V_O5_	6.31	6.14	5.57	4.93
	V_O6_	6.47	6.47	5.94	5.32
O-oct	V_O1_	5.17	5.12	4.72	4.35
	V_O2_	5.96	6.05	5.43	4.93
M-oct	V_O1_	5.26	5.35	4.85	4.51
					Unit: eV

Here, we focus on the O atom with the minimum *E*_Ovac_ value. One type of O atom is exposed on the (100), (110), and M-oct surfaces (Figure 2(a,b,e), respectively). However, the stepped (100) surface has O atoms with different environments. The calculated *E*_Ovac_ values for the different O atoms show that the O atom on the step-edge (V_O1_ in Figure 2(c)) is the easiest to remove. The O-oct surface has two types of surface O atoms (Figure 2(d)). The calculation results indicate that the *E*_Ovac_ value of the O atom with the lowest coordination number (V_O1_ in Figure 2(d)) is smaller than the *E*_Ovac_ value of the other O atom.

When the BG was applied to explain the trend of the *E*_Ovac_ values in the present study, the *R*^2^ value was calculated to be 0.73 (Figure S1). This result shows that the BG is a useful descriptor for interpreting the *E*_Ovac_ values of various surfaces, not just the perfect surface, which is physically reasonable. The valence-band maximum (VBM) is made up mostly of the crystal orbital of the O atom (Figure 3). By contrast, the contribution of the alkaline-earth metal atom to the conduction-band minimum (CBM) increases compared with its contribution to the VBM. This result for the DOS is consistent with the general trend for most metal oxides. The VBM energy is expected to be correlated with the stability of an O atom on a metal oxide surface. When O atoms are removed from the metal oxide surface, the excess electrons are considered to be transferred to a level above the VBM. In most cases, the excess electrons are expected to be transferred to the CBM; the CBM energy modulates the stability of the remaining excess electrons via the formation of O vacancies. This effect is why the BG (i.e. the difference between the VBM and CBM) shows a good correlation with the *E*_Ovac_ value. From a chemical perspective, the hardness of the metal oxide surface can explain the trend of the *E*_Ovac_ values. The hardness is defined as one-half of the BG. Thus far, the hardness has been commonly used to explain phenomena observed in atomic and molecular systems [111,112]. The present study shows that the concept of hardness is applicable to complicated surface systems, which supports the findings of our previous work [93].

We here examine the relationship between the BG and *E*_Ovac_ values in detail. As a general trend, the *E*_Ovac_ value decreases with decreasing BG. The BG values of alkaline-earth metal oxides tend to decrease with increasing atomic number of the alkaline-earth metal: 6.27, 6.17, 5.60, and 5.09 eV for the MgO, CaO, SrO, and BaO (100) surfaces, respectively (Table 2). One of the reasons for this trend is the difference in the atomic radii of the alkaline-earth metals. Because the ionic radius increases in conjunction with increasing period [113], the Coulomb force between an alkaline-earth metal and an O atom is expected to increase in the order Mg > Ca > Sr > Ba. In their discussion of the different surface reactivities of MgO and CaO surfaces, Pacchioni et al. noted that an O atom on the surface of an alkaline-earth metal oxide is stabilized by the Madelung potential of the ionic crystal [114]. Thus, the electrostatic interaction between an alkaline-earth metal and a surface O atom might strongly affect the stability of the O atom. Another reason for the aforementioned trend is the difference in the ionization potentials for the alkaline-earth metals. The ionization potential of the alkaline-earth metals decreases from top to bottom within the periodic table [115], which reduces the interaction between the 2*p* atomic orbital of the O atom and the *s*-type atomic orbital of the alkaline-earth metal. These differences in orbital interaction result in differences in the BG, which can explain the aforementioned trend of the *E*_Ovac_ values.

We also compared the dependence of the *E*_Ovac_ values on the surfaces of each alkaline-earth metal oxide. The results in Table 2 show that the minimum *E*_Ovac_ value of the minor surfaces—i.e. the (110), stepped (100), O-oct, and M-oct surfaces – is smaller than that of the (100) surface. The maximal change of the *E*_Ovac_ value on the basis of the difference in the surface structure is ~1.0 eV for all of the alkaline-earth metal oxides: for example, the difference in the *E*_Ovac_ value between the (100) surface and the (110) surface of MgO is 1.10 eV. In the case of CaO, the *E*_Ovac_ value of the O-oct surface is smaller than that of the (100) surface by 1.06 eV. This result implies that different surface configurations of alkaline-earth metal oxides can lead to major differences in their catalytic ability.

### 3.3. Electronic structure of the surface

**Figure 4.**
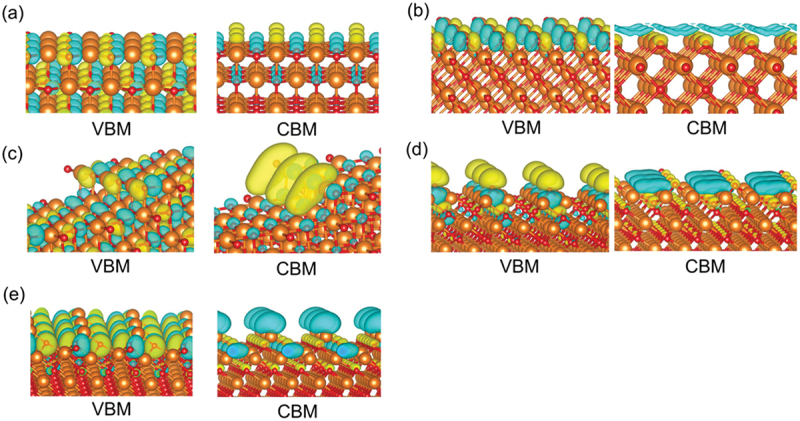
Crystal orbitals of the VBM and CBM for MgO surfaces: (a) (100) surface, (b) (110) surface, (c) stepped (100) surface, (d) O-oct surface, and (e) M-oct surface. The orange and red spheres represent Mg and O atoms, respectively.

Conceivably, the crystal orbital near the Fermi level of alkaline-earth metal oxides strongly affects their catalytic activity, as assumed under the concept of frontier molecular orbital theory. Figure S3 shows the crystal orbitals of the VBM and CBM for the five types of surfaces of all of the investigated alkaline-earth metal oxides. Figure 4 shows the crystal orbitals of MgO as an example.

Although the VBM of the (100) surface (Figure 4(a)) mainly consists of oxygen 2*p* orbitals (which exhibit an antibonding interaction with each other) corresponding to the bulk valence band, the CBM originates from the 3*s* and 3*p* states of the alkaline-earth metals. This picture is in agreement with the findings of a previous report [116]. The emergence of the step defects induces a drastic change of the crystal orbitals, as shown in Figure 4(c). The VBM of the stepped (100) surface has oxygen 2*p* orbitals on the step-edge part, and these orbitals extend from the surface. In the case of the CBM, orbitals are localized at the alkaline-earth metal on the step-edge part. These results for crystal orbitals imply that both the Lewis basicity and the Lewis acidity of the stepped (100) surface are stronger than those of the (100) surface.

The VBM of the (110) surface (Figure 4(b)) shows an antibonding interaction between oxygen 2*p* orbitals on the outermost surface; this interaction might be responsible for this surface exhibiting the smallest BG among the surfaces under consideration. However, orbitals localized at the space between alkaline-earth metals on the outermost surface in the CBM; this space is where oxygen is present in the bulk phase.

Orbitals extend from the octopolar (111) surfaces for the VBM of the O-oct surface and the CBM of M-oct surface. These localized orbitals are derived from the three-fold coordinated atoms on these surfaces. These results imply a high Lewis basicity of the O-oct surface and a high Lewis acidity of the M-oct one. For the other orbitals of the octopolar (111) surfaces, the CBM of the O-oct surface mainly consists of the localized orbital over the cavity. The VBM of the M-oct surface shows the antibonding interaction between the 2*p* orbitals of **O**_**4c**_.

### 3.4. Adsorption configuration and regression analysis with the objective of developing a promising catalyst

#### 3.4.1. Adsorption of a methyl radical

**Figure 5.**
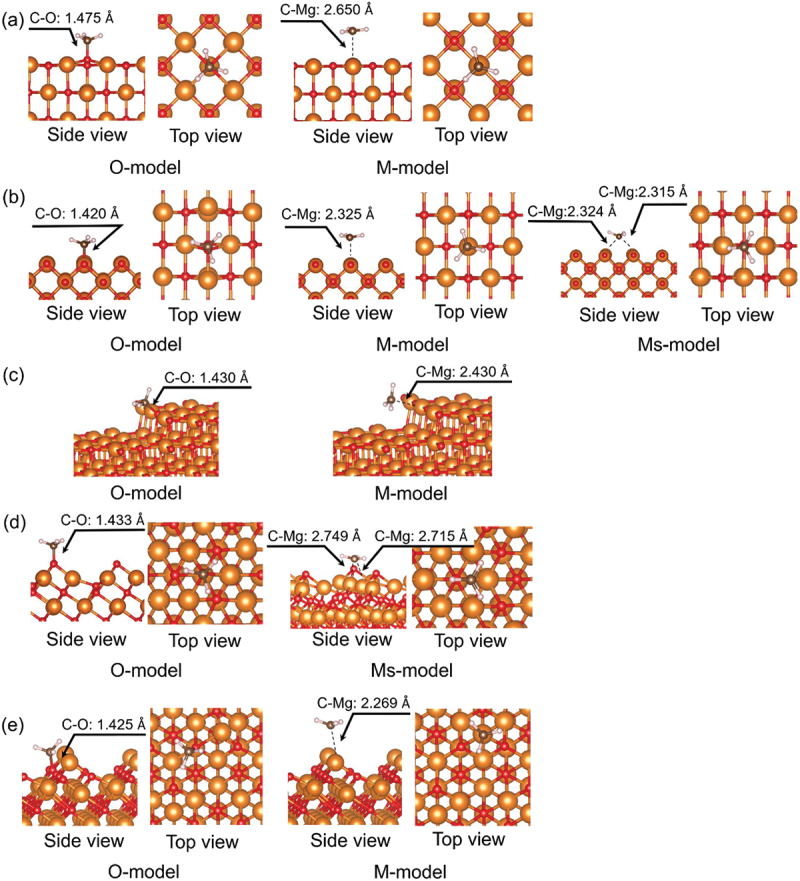
Adsorption structures for a methyl radical on MgO surfaces: (a) (100) surface, (b) (110) surface, (c) stepped (100) surface, (d) O-oct surface, and (e) M-oct surface. The orange, red, brown, and white spheres represent Mg, O, C, and H atoms, respectively.

As mentioned in Introduction, the bonding strength between the methyl radical and metal oxide surfaces is associated with the catalyst poisoning. To estimate the energies of *E*_CH3_ads_, we need to explore the adsorption configurations of a methyl radical on the alkaline-earth metal oxide surfaces under consideration. Figure S4 illustrates the most stable adsorption configuration for each surface of the alkaline-earth metal oxide surfaces. The adsorption energies for these configurations are listed in Table 3. The configuration on the MgO surface is depicted in Figure 5 as an example.

**Table 3.** Computed adsorption energies of methyl radical (*E*_CH3_ads_) on alkaline-earth metal surfaces.

		MgO	CaO	SrO	BaO
(100)	O-model	0.75	0.04	−0.48	−1.20
	M-model	−0.35	−0.32	−0.29	−0.27
(110)	O-model	−0.88	−1.59	−1.90	−2.13
	M-model	−0.16	−0.42	−0.42	−0.51
	Ms-model	−0.33	−0.65	−0.68	−0.76
stepped (100)	O-model	−0.57	−1.07	−1.51	−1.92
	M-model	−0.64	−0.54	−0.39	−0.48
O-oct	O-model	−1.02	−1.59	−1.88	−1.93
	Ms-model	−0.50	−0.68	−0.80	−0.69
M-oct	O-model	−0.96	−1.21	−1.54	−1.63
	M-model	−0.68	−0.16	−0.20	−0.35
					Unit: eV

Methyl radicals primarily adsorb at two types of sites: the O site (O-model) and the M site (M-model). For the MgO (100) surface, shown in Figure 5(a), the M-model is more stable than the O-model by 1.1 eV. Although the CaO (100) surface shows the same tendency as the MgO(100) surface, the SrO(100) and BaO(100) surfaces show a preference for the O-model (Table 3). Notably, the *E*_CH3_ads_ values of the O-models become increasingly negative with increasing atomic number of the alkaline-earth metal: 0.75, 0.04, −0.48, and − 1.21 for MgO, CaO, SrO, and BaO, respectively. This trend is consistent with that observed for the adsorption of an H atom (section 3.4.4). However, those of the M-models for each metal are approximately constant: −0.35, −0.31, −0.29, and − 0.27 for MgO, CaO, SrO, and BaO, respectively. Based on the stability comparison between the O-model and the M-model, the most stable *E*_CH3_ads_ values for each oxide are −0.35 eV for MgO, −0.31 eV for CaO, −0.48 eV for SrO, and −1.21 eV for BaO, respectively. These results showed that increasing the atomic number of the alkaline-earth metal leads to a more negative *E*_CH3_ads_ value, indicating stronger adsorption.

A similar trend is also observed in the *E*_CH3_ads_ values for the stepped (100) surface (Figure 5(c)); however, the methyl radicals form stronger bonds with this surface compared to the (100) surface. A comparison of *E*_CH3_ads_ between the *M*- and O-models reveals that, for MgO, the values are almost identical; however, for the other alkaline-earth metal oxides, the O-model is energetically more favorable. Additionally, when we examined the adsorption of a methyl radical onto the terraced part of the stepped (100) surface, we found that adsorption on the step-edge site was energetically superior. This result is consistent with the findings that this site has the lowest *E*_Ovac_ value, indicating a preference for adsorbate to bind to the O atom with the minimum *E*_Ovac_.

For the (110) surface, we compare the *E*_CH3_ads_ values for three models: the O-model, M-model, and the Ms-model, in which the methyl radical interacts with several metal atoms on the surface (Figure 5(b)). In the Ms-model, a methyl radical is positioned between two metal atoms, at a site where large electron densities are observed in the CBM (Figure 4(b)). Among the three models, the O-model exhibits the most stable *E*_CH3_ads_ value for all of the investigated alkaline-earth metal oxides.

We confirmed two stable configurations for the O-oct surface: the O-model and the Ms-model (Figure 5(d)), where the former is a more stable configuration than the latter for all of the investigated alkaline-earth metal oxides. In the case of the M-oct surface, the methyl radical prefers the O-site to the M-site (Figure 5(e)).

**Figure 6.**
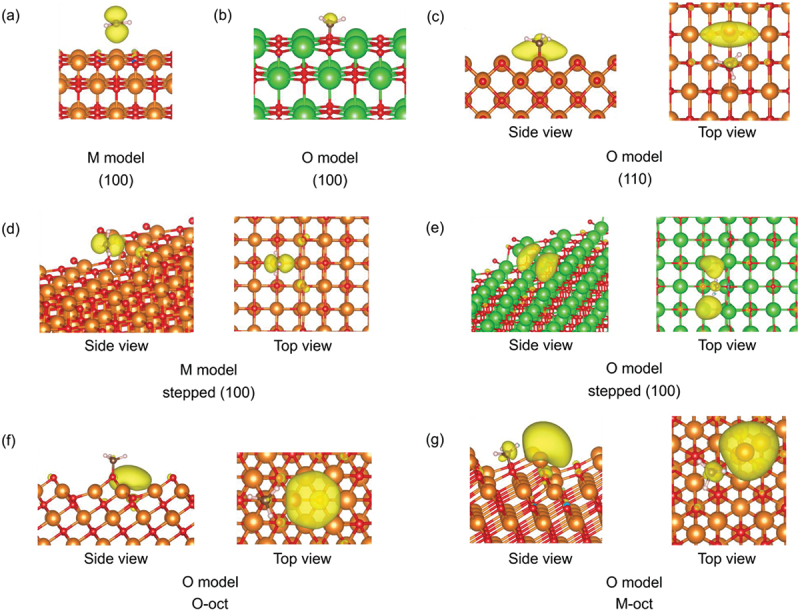
Spin-density isosurfaces, in yellow, on MgO and BaO surfaces with a methyl radical: (a) M-model of the MgO (100) surface, (b) O-model of the BaO (100) surface, (c) O-model of the MgO (110) surface, (d) M-model of the MgO stepped (100) surface, (e) O-model of the BaO stepped (100) surface, (f) O-model of the MgO O-oct surface, and (g) O-model of the MgO M-oct surface. The orange, green, red, brown, and white spheres represent Mg, Ba, O, C, and H atoms, respectively.

Figure 6 illustrates the spin density on the surface of MgO and BaO with an adsorbed methyl radical. Basically, the spin densities in the M-model are localized on the methyl radical (Figure 6(a,d)). However, in the case of the O-model, spin densities are localized on the nearest metal site, similar to the CBM (Figure 4).

According to the literature, the correlation between *E*_CH3_ads_ and *E*_Ovac_ can be used to identify high-performance OCM catalysts [91]. The *R*^2^ value of the correlation between the *E*_CH3_ads_ and the *E*_Ovac_ value, 0.84, is higher than that of the correlation between the *E*_CH3_ads_ and the BG value, 0.62 (Figure S5 (a) and (c)); hence, the *E*_Ovac_ value can explain the trend of the *E*_CH3_ads_ value.

#### 3.4.2. Effect of the temperature on E_CH3_ads_

**Figure 7.**
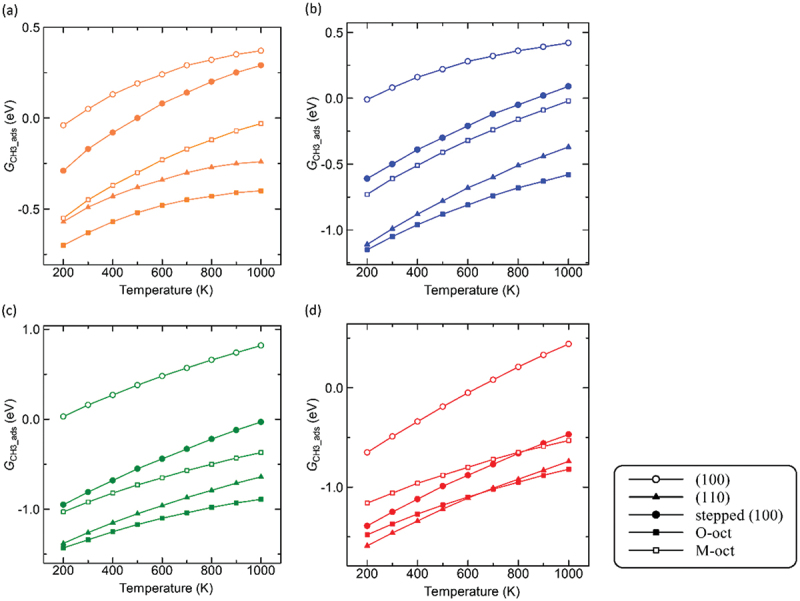
*G*_CH3_ads_ values as a function of temperature: (a) MgO, (b) CaO, (c) SrO, and (d) BaO.

The OCM mechanism might be strongly influenced by entropic effects. In this section, we explore the impact of temperature on the interaction between the methyl radical and metal oxide surfaces based on the Gibbs free energy. While our current focus has been on entropic effects, kinetic factors, which could be analyzed through approaches such as microkinetic analysis or transition state theory, may also play a crucial role in gaining a more comprehensive understanding of the temperature effects. These aspects will be explored in future studies. Figure 7 illustrates the relationship between the Gibbs free energy of the CH_3_ adsorption (*G*_CH3_ads_) value and the temperature in the range from 200 to 1000 K. It shows that an increase in temperature increases the *G*_CH3_ads_ value toward the positive direction, which indicates that the interaction between the methyl radical and the surfaces becomes weak with increasing temperature. As previously mentioned, OCM catalyst should resist poisoning by the methyl radical. Higher temperatures facilitate the desorption of this radical from the surfaces. Figure S8 depicts the relationship between the C – H activation Gibbs free energy for the CH_3_ production (*G*_CH3_act_) and the temperature. The *G*_CH3_act_ values decrease with increasing temperature, although the magnitude of change in the *G*_CH3_act_ values is lower compared with the magnitude of the change in the *G*_CH3_ads_ values.

For all of the alkaline-earth metal oxides investigated, the (100) surfaces reach a the *G*_CH3_ads_ value above 0.0 eV at lower temperatures than other investigated surfaces. Especially, the SrO (100) surface exhibits positive *G*_CH3_ads_ values starting at just 200 K, indicating minimal poisoning from methyl radicals during the OCM reaction. Judging from the high *G*_CH3_ads_ value, the SrO (100) surface is expected to be the ideal surface for the OCM reaction.

Although the (100) surfaces appear to be promising on the basis of the trend of the *G*_CH3_ads_ values at increasing temperature, these surfaces require a large activation energy to break the C – H bond. Figure 7 reveals that most of the *G*_CH3_ads_ values for the peculiar surfaces are negative but become positive as temperature increases. Interestingly, the *G*_CH3_ads_ value of the MgO stepped (100) surface changes from a negative to a positive value between 500 and 600 K, while for the CaO stepped (100) surface, this shift occurs at higher temperature between 800 and 900 K. This suggests that, at higher temperatures, the stepped (100) surfaces might be more promising catalysts compared to the (100) surfaces, due to their capability to release the methyl radical.

#### 3.4.3. H abstraction of the CH_4_ molecule

Our investigation into the effect of the temperature on *E*_CH3_ads_ revealed that the stepped (100) surfaces are promising candidates for OCM catalysts. This is due not only to their resistance to the surface poisoning by the methyl radical but also to their ability to activate the C-H bond of a CH_4_ molecule under a mild condition, which will be discussed further in this section.

**Figure 8.**
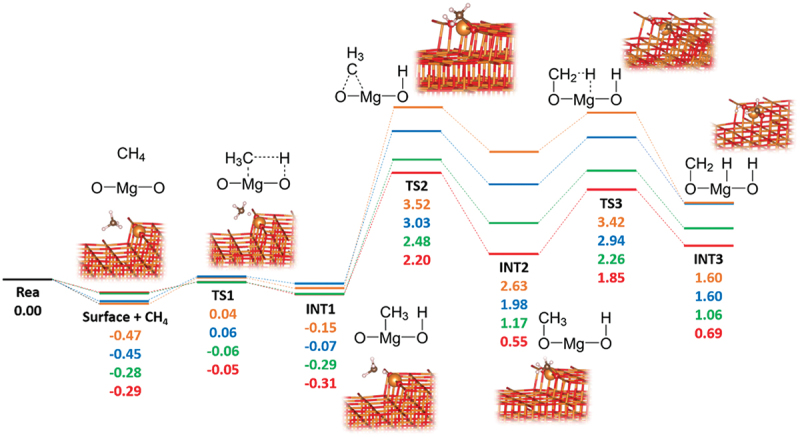
Reaction path of H abstraction of the CH_4_ molecule and methyl radical to stepped (100) surfaces of alkaline-earth metals oxides under consideration. The paths for MgO, CaO, SrO, and BaO are colored red, blue, green, and red, respectively.

We explored the H abstraction from the CH_4_ on the stepped (100) surfaces of metal oxides. Figure 8 illustrates the reaction paths for each oxide. On these surfaces, the energies required to abstract a hydrogen atom from the CH_4_ molecule were calculated to be 0.51 eV for both MgO and CaO, 0.22 eV for SrO, and 0.24 eV for BaO. This result indicates that the unique structure of the stepped (100) surface facilitates the cleavage of C-H bond of the CH_4_ molecule. These values tend to decrease with increasing atomic number of the alkaline-earth metal, which is in agreement with the trend of the *E*_Ovac_ values (Table 2). This trend can be interpreted on the basis of the Madelung potential of each alkaline-earth metal oxide reported by Pacchioni et al. [114], as discussed in section 3.2. A small value of the Madelung potential indicates instability of the O atom. Pacchioni et al. [114] reported that CaO exhibits greater basicity and reactivity than MgO because the Madelung potential is smaller in the former than in the latter. From a different perspective, Abdel Halim et al. showed that the surface basicity increases in the order MgO < CaO < SrO < BaO [117]. The trend of the H abstraction energies obtained in the present work is supported by these previously reported results. As mentioned in section 3.4.1, the difference in orbital interaction between alkaline-earth metals and surface O atoms may influence the stability of surface O atoms, affecting the H abstraction energy.

We further investigated H abstraction from the adsorbed methyl radical to assess whether the CH_3_ species undergoes over-oxidation on these surfaces (Figure 8). After the initial H abstraction from the CH_4_ molecule to the step (100) surface, the methyl radical prefers the M site (**INT1**). However, the methyl radical needs to migrate from the M site to neighboring O one for the H abstraction from the CH_3_ species (**TS2**). The energies required for this migration were calculated to be 3.67, 3.10, 2.77, and 2.51 for MgO, CaO, SrO, and BaO, respectively, suggesting that the over-oxidation is suppressed under mild condition and the desorption of the methyl radical is favored.

Two mechanisms for OCM on metal oxides are known: the ‘hydrogen abstraction’ and the ‘two-site four-centered’ mechanism [118,119]. The former mechanism involves surface O atoms in the H abstraction, generating surface O-H species and CH_3_ radical, while the latter results in M-CH_3_ and O-H species. Although the (100) surfaces promote the cleavage of C-H bond of CH_4_ via the ‘hydrogen abstraction’ mechanism [91], the step (100) surfaces are found to utilize the ‘two-site four-centered’ mechanism, as supported by our DFT calculations and corroborated by the previous report [52]. Here, it should be noted that, further oxidation of CH_3_ species is difficult on this step surfaces as mentioned above, which indicates that the process of the C_2_ production is likely the result of coupling between methyl radicals that have desorbed from the surface. This finding helps us to identify promising catalysts for OCM using a ‘pseudo-steady-state approach’.

#### 3.4.4. Adsorption of an H atom

**Figure 9.**
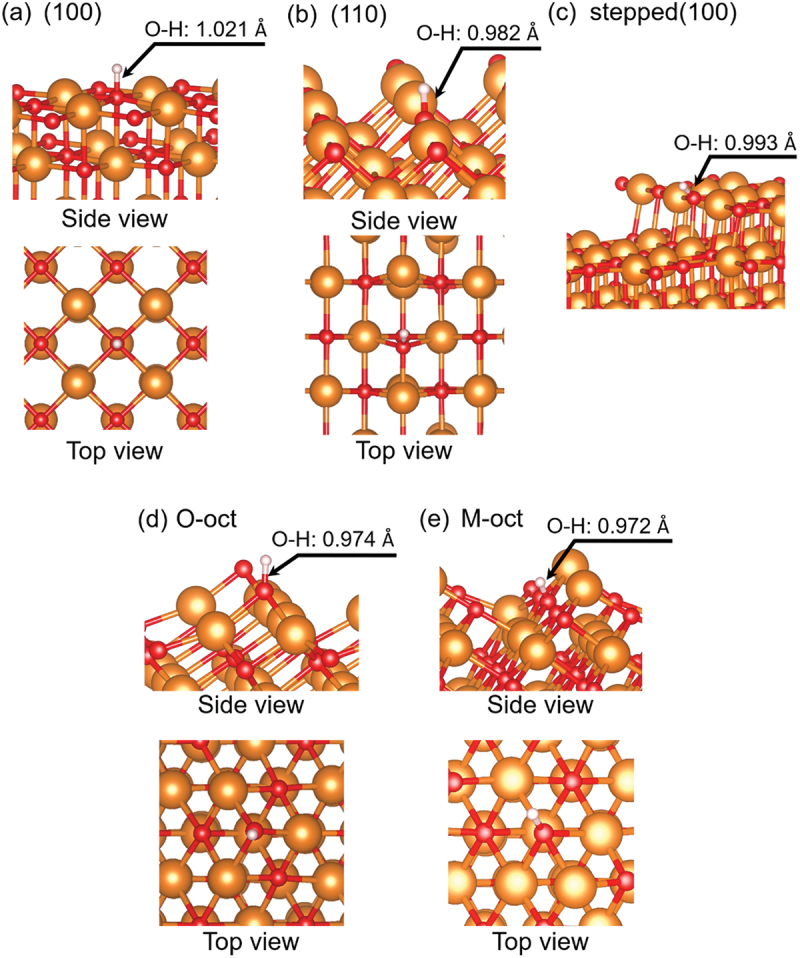
Adsorption structures for a H atom on MgO surfaces: (a) (100) surface and (b) stepped (100) surface. The orange, red, and white spheres represent Mg, O, and H atoms, respectively.

**Figure 10.**
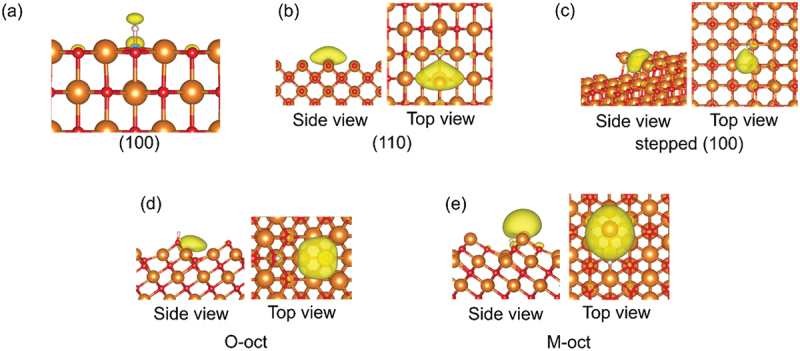
Spin-density isosurfaces, in yellow, on each MgO surface with a H atom: (a) (100) surface, (b) (110) surface, (c) stepped (100) surface, (d) O-oct surface, and (e) M-oct surface. The orange, red, and white spheres represent Mg, O, and H atoms, respectively.

Following the analysis in the section 3.4.1, which details *E*_CH3_ads_, this section focuses on H atom adsorption to estimate *E*_act_ across the alkaline-metal oxide surfaces. Figure S6 illustrates the most stable adsorption configuration for each surface of the alkaline-earth metal oxide surfaces. The adsorption energies for these configurations are listed in Table 4. Figures 9 and 10 illustrate the configurations and spin densities on the MgO surface as an example.

**Table 4.** Computed adsorption energies of a H atom (*E*_H_ads_) on alkaline-earth metal surfaces.

	MgO	CaO	SrO	BaO
(100)	−1.56	−2.17	−2.64	−3.07
stepped (100)	−2.83	−3.15	−3.53	−3.71
				Unit: eV

On all examined surfaces, H atoms preferentially adsorb not to the metal sites but to the O sites with the lowest coordination numbers. This result is in agreement with the results of previous works [52,91]. Notably, H atoms adsorb specially to the O atoms with the lowest *E*_Ovac_ value within each surface model, especially evident on the stepped (100) surface as detailed in section 3.2. The stepped (100) surface contains several **O**_**5c**_ atoms in various environments in addition to the **O**_**4c**_ atom in the step-edge part. Compared to the **O**_**5c**_ atoms, the **O**_**4c**_ atoms, which have lower coordination number and highlighted by the crystal orbitals of the VBM and CBM (Figure 4), are better sites for the H adsorption. When it comes to the O-oct surface, there are two types of surface O atoms: **O**_**3c**_ and **O**_**5c**_ (Figure 1). The H atom prefers the former site to the later site as shown in Figure 9(d). On the M-oct surface, **O**_**4c**_ site, of which the coordination number is lower than that of **O**_**6c**_ atom in bulk, acts as the adsorption site (Figure 9(e)).

As listed in Table 4, the *E*_H_ads_ values of unique surfaces are lower than those of the (100) surface for all of the investigated alkaline-earth metal oxides; as an example, the *E*_H_ads_ energies of the MgO (100), (110), stepped (100), O-oct, and M-oct surfaces are − 1.56, −2.83, −2.99 eV, −2.98, and −3.13 eV, respectively. This result shows that the difference in surface morphology strongly affects the adsorption ability. As shown in Figure 10, the unpaired electron of the H atom is transferred to the nearest metal site except for the (100) surface, indicating that easy acceptance of electrons on the metal site is important to increase the *E*_H_ads_ values negatively. In the case of the (100) surface, the electron transfer is not observed (Figure 10(a)), because of the wide BG, which makes the electron transfer of the H atom to the CBM difficult. A comparison of the *E*_H_ads_ energies among the alkaline-earth metal oxides shows that they increase negatively with increasing atomic number of the metal: −1.56, −2.17, −2.64, and −3.07 eV for the MgO, CaO, SrO, and BaO (100) surfaces, respectively (Table 4). This trend can be interpreted on the basis of the Madelung potential of each alkaline-earth metal oxide, which mentioned in section 3.4.3.

Figures S5 (b) and (d) show the correlation between the *E*_Ovac_ or BG and the *E*_H_ads_. The *R*^2^ value for the *E*_Ovac_ value, 0.84, is higher than that (0.62) for the BG, which indicates that the *E*_Ovac_ value is a better descriptor than the BG for predicting the *E*_H_ads_ value.

#### 3.4.5. Correlation between E_act_ and several variables: E_CH3_ads_, E_Ovac_, and BG

**Figure 11.**
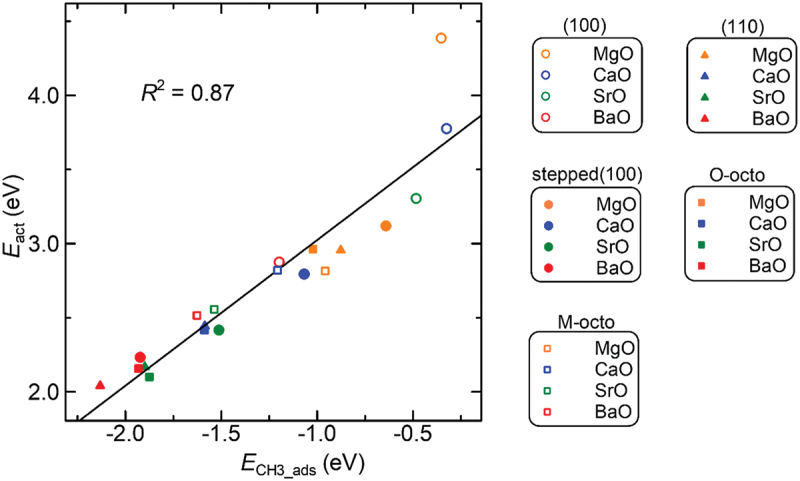
Correlation between *E*_act_ and *E*_CH3_ads_. The line represents a regression line. The open circles, filled circles, filled triangles, filled squares, and open squares represent the (100), stepped (100), (110), O-oct, and M-oct surfaces, respectively. The points corresponding to MgO, CaO, SrO, and BaO in this figure are orange, blue, green, and red, respectively.

Thus far, we have discussed the adsorption configurations of a H atom and a methyl radical on the alkaline-earth metal oxide surfaces. Here, we estimate the *E*_act_ values using the Brønsted – Evans – Polanyi relationship described in section 2. These values are compiled in Table 5. A comparison among the alkaline-earth metal oxides shows that *E*_act_ values decrease with increasing atomic number of the metal: 4.39, 3.78, 3.30, and 2.87 eV for the MgO, CaO, SrO, and BaO (100) surfaces, respectively. We confirmed in section 3.3.1 and 3.3.4 that the *E*_H_ads_ and *E*_CH3_ads_ values negatively increase in the series MgO < CaO < SrO < BaO. These results reveal that a trade-off relationship exists between the *E*_act_ and the *E*_H_ads_ or *E*_CH3_ads_ values, as mentioned in the Introduction. This trend is also supported by the results in Figure 11, which show a linear correlation between the *E*_act_ value and the *E*_CH3_ads_ value, where the *R*^2^ value was calculated to be 0.81. Irrespective of the surface type, an increase in the *E*_CH3_ads_ value leads to an increase in the *E*_act_ value.

**Table 5.** Computed activation energies of the C-H bond cleavage in CH_4_ (*E*_act_) on alkaline-earth metal surfaces.

	MgO	CaO	SrO	BaO
(100)	4.39	3.78	3.30	2.87
(110)	2.96	2.44	2.17	2.04
stepped (100)	3.12	2.79	2.42	2.23
O-oct	2.96	2.42	2.10	2.16
M-oct	2.82	2.82	2.56	2.52
				Unit: eV

An efficient OCM catalyst should demonstrate two main properties: (1) the ability to easily break the C – H bond and (2) durability against poisoning by the methyl radical. These properties are characterized by the *E*_act_ value and the *E*_CH3_ads_ value, respectively. To obtain value-added chemicals via the OCM, a surface with a high *E*_CH3_ads_ value and a low *E*_act_ value is preferred. From these perspectives, the correlation diagram in Figure 11 is expected to reveal a beneficial catalyst for the OCM. In addition to these two properties, it is crucial to avoid over-oxidation under oxidative conditions. As demonstrated in section 3.4.3, the alkaline-earth metal oxide surfaces are less prone to over-oxidation because the second C – H bond scission is not facile. This characteristic further supports the potential of these surfaces as efficient and selective catalysts for the OCM reaction.

As we can see from considerable attention devoted to the OCM mechanism of MgO surfaces, high temperatures are well known to be required for this surface to activate the C – H bond of CH_4_. However, as mentioned in the Introduction, the irregular sites on a surface, which are not present in the most stable surface without defects, play an important role in activating an adsorbate, as supported by experimental and computational studies thus far [5,48,62–66]. The *E*_act_ values of the step (100) surface of alkaline-earth metal oxides are substantially smaller than the value of the (100) surface. In addition, the step (100) surfaces of the metal oxides, especially MgO, exhibit higher *E*_CH3_ads_ values, indicating that these surfaces are less likely to be poisoned by the methyl radical; therefore, the step (100) surfaces can activate the C – H bond, with good selectivity for the OCM reaction. The properties deduced through calculations in the present report indicate that the peculiar surfaces of MgO strongly contribute to the formation of value-added chemicals under the OCM reaction.

Finally, we here focus on the SrO (100) surface. The *E*_CH3_ads_ value for the SrO (100) surface suggests that, compared with the MgO (100) surface, it is slightly more easily poisoned by adsorption of a methyl radical: *E*_CH3_ads_ = −0.35 and −0.48 eV for MgO and SrO, respectively. This result likely arises from the difference in the most stable configuration of the methyl radical on these surfaces, as mentioned in section 3.4.2; the SrO (100) surface prefers the O-model, whereas the MgO (100) surface prefers the M-model. Because the *E*_CH3_ads_ value of the SrO (100) surface is relatively larger than that of each surface under consideration, this surface is less likely to be poisoned by the methyl radical. The *E*_act_ value shows that the SrO (100) surface is superior to the MgO (100) surface with respect to the ability to break the C – H bond. This trend can be explained in terms of the Madelung potential, as mentioned above; SrO exhibits greater basicity and reactivity than MgO because the Madelung potential decreases with increasing atomic period. From these investigations of the *E*_CH3_ads_ and *E*_act_ values, the SrO (100) surface is predicted to be a useful catalyst for the OCM. This result supports those of Carreiro *et al*., who experimentally showed that SrO exhibits the highest C_2_ selectivity among alkaline-earth metal oxide compounds [120]. Here, we note that other factors, such as the interaction between the surface and water vapor, can adversely affect the selectivity.

## 4. Conclusions

We used first-principles calculations to investigate the ability of five types of surfaces (i.e. the (100), (110), stepped (100), O-oct, and M-oct surfaces) of alkaline-earth metal oxides (i.e. MgO, CaO, SrO, and BaO) to catalyze the OCM reaction. First, we determined the O atom with the minimum *E*_Ovac_ value on each surface and found that the *E*_Ovac_ value of the O atom with the lowest coordination number was the smallest. A comparison of the *E*_Ovac_ values among the surfaces in each alkaline-earth metal oxide showed that the minimum *E*_Ovac_ value of each unique surface (i.e. (110), stepped (100), O-oct, and M-oct surfaces) was lower than that of the (100) surface.

The most stable configurations of the H atom and the methyl radical showed that these adsorbates are likely to be adsorbed onto the O atom with the minimum *E*_Ovac_ value on each surface. The *R*^2^ values of the correlations between the *E*_Ovac_ and the *E*_H_ads_ values, and those of the correlations between the *E*_Ovac_ and the *E*_CH3_ads_ values, indicated that the *E*_Ovac_ value can explain the trend of these adsorption energies.

An ideal OCM catalyst should demonstrate two main properties: (1) the ability to break the C – H bond easily and (2) durability toward poisoning by the methyl radical. From this perspective, the correlation between the *E*_act_ and the *E*_CH3_ads_ values revealed several surfaces that are preferred as OCM catalyst surfaces. In particular, taking the entropic effect on the OCM mechanism into consideration, we identified several candidates as promising OCM catalysts: stepped (100) surfaces and SrO (100) surfaces. The utility of these surfaces has been supported by several researches thus far. As discussed in section 3.4.3, these surfaces are less prone to over-oxidation because the second C – H bond scission is not facile, further enhancing their potential as efficient and selective OCM catalysts.

## Supplementary Material

Supplemental Material
