# Supplementary material for: Effect of the surface morphology of alkaline-earth metal oxides on the oxidative coupling of methane
Source: Sci Technol Adv Mater. 2024 Dec 20;26(1):2435801. doi: 10.1080/14686996.2024.2435801 (PMC11703441; doi:10.1080/14686996.2024.2435801)
Supplement: Supplemental Material [file TSTA_A_2435801_SM6383.docx]

Supporting Information for:

The Effect of the Surface Condition of Alkaline-Earth Metal Oxides on the Oxidative Coupling of Methane

Nobutsugu Hamamoto^1^, Takakazu Kawahara^2^, Ryoto Hagiwara^2^, Kohei Matsuo^2^, Kodai Matsukawa^2^, Yoyo Hinuma^3^, Takashi Toyao^4^, Ken-ichi Shimizu^4^ and Takashi Kamachi^2^

^1^ Department of Applied Chemistry, Faculty of Engineering, Sanyo-Onoda City University, Sanyo-Onoda, Yamaguchi 756-0884, Japan

^2^ Department of Life, Environment and Applied Chemistry, Fukuoka Institute of Technology, Fukuoka, Wajiro-higashi, Higashi-ku, Fukuoka 811-0295, Japan

^3^ Department of Energy and Environment, National Institute of Advanced Industrial Science and Technology (AIST), 1-8-31 Midorigaoka, Ikeda, Osaka 563-8577, Japan

^4^ Institute for Catalysis, Hokkaido University, Sapporo, Hokkaido 001-0021, Japan


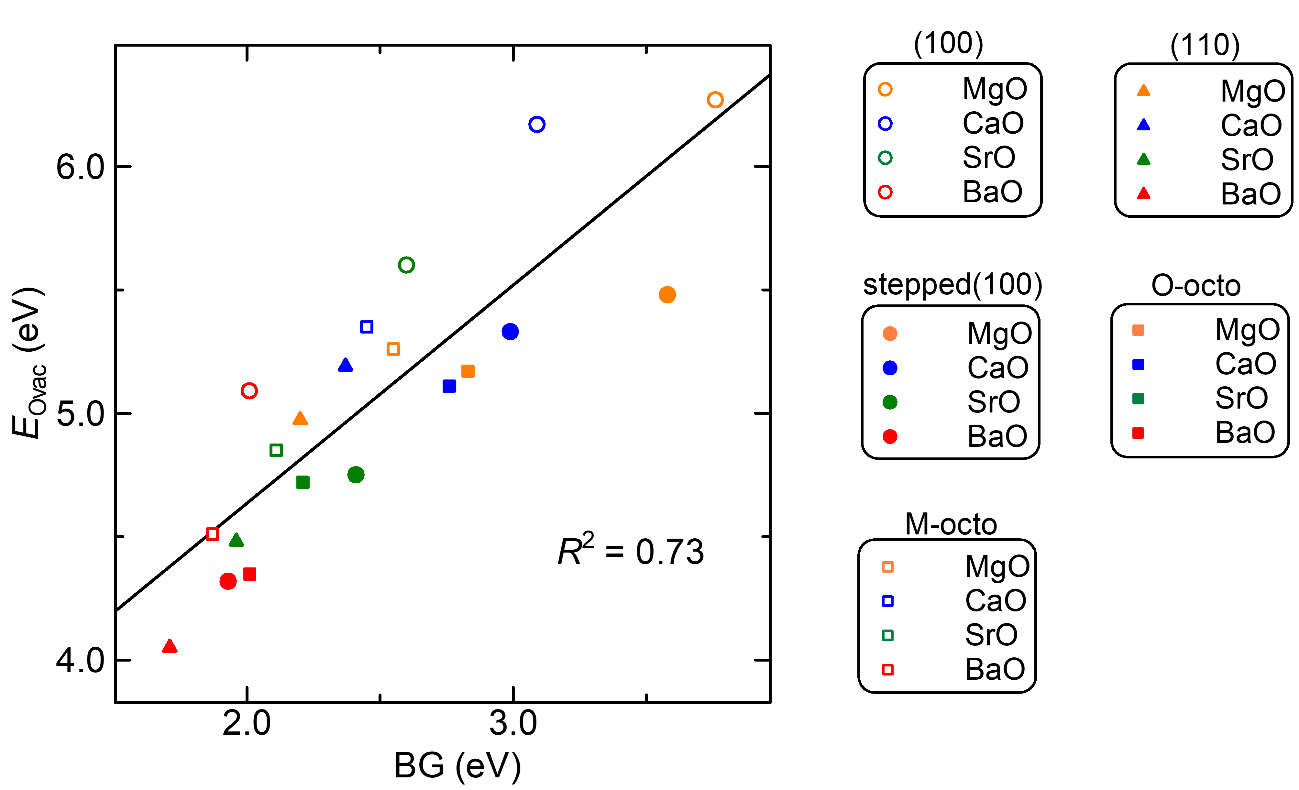


**Figure S1.** *E*_Ovac_ as a function of BG for each surface. The line represents a regression line. The open circles, filled circles, filled triangles, filled squares, and open squares represent the (100), stepped (100), (110), O-oct, and M-oct surfaces, respectively. The points representing MgO, CaO, SrO, and BaO in this figure are orange, blue, green, and red, respectively.

MgO

CaO

SrO

BaO

**Figure S2.** Total and partial DOS plots for MgO, CaO, SrO and BaO surfaces under consideration in this study. The black line shows the total DOS, and red and blue lines represents the partial DOS of the oxygen and alkaline-earth metal atoms, respectively. The DOS plots multiplied by several times are also depicted for the neighborhood of CBM. The energy values were determined from the Fermi level.

MgO


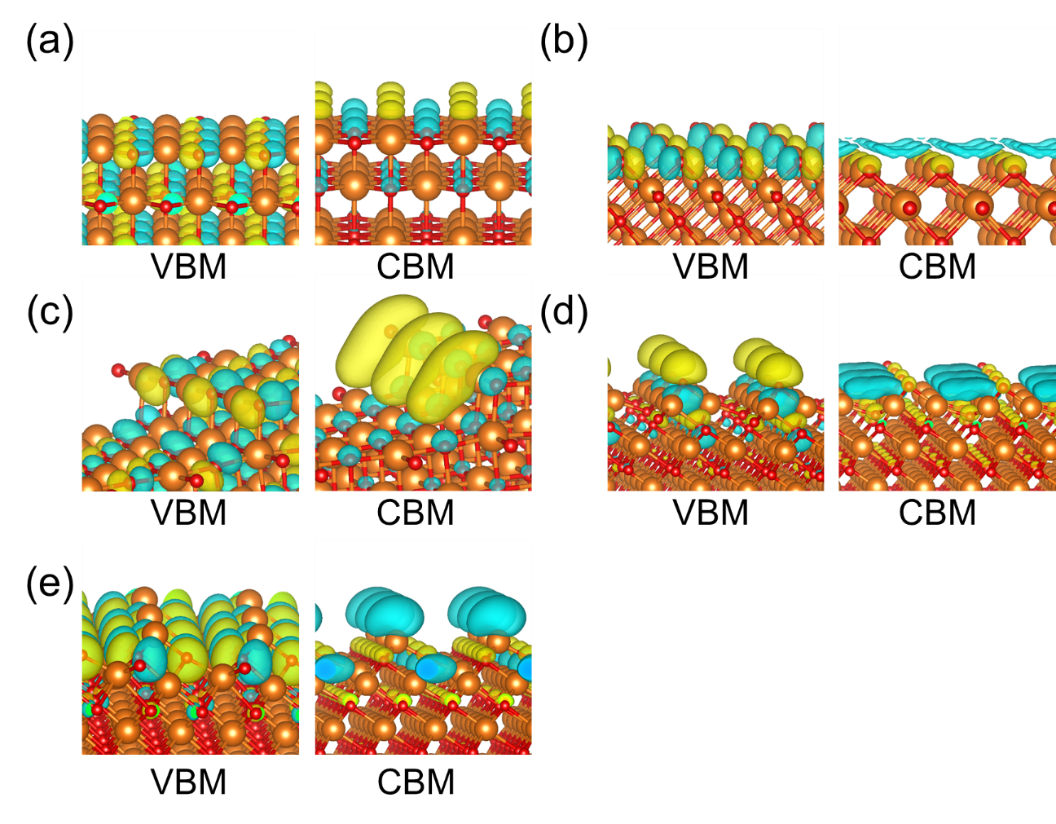


CaO


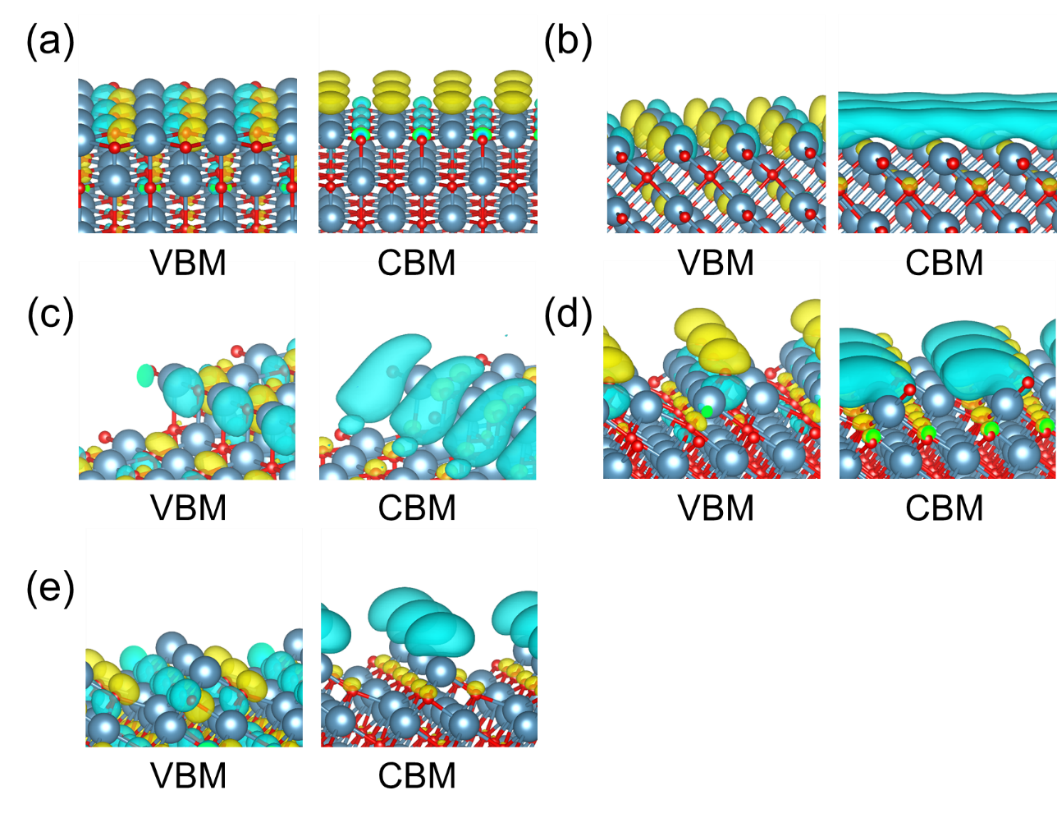


SrO


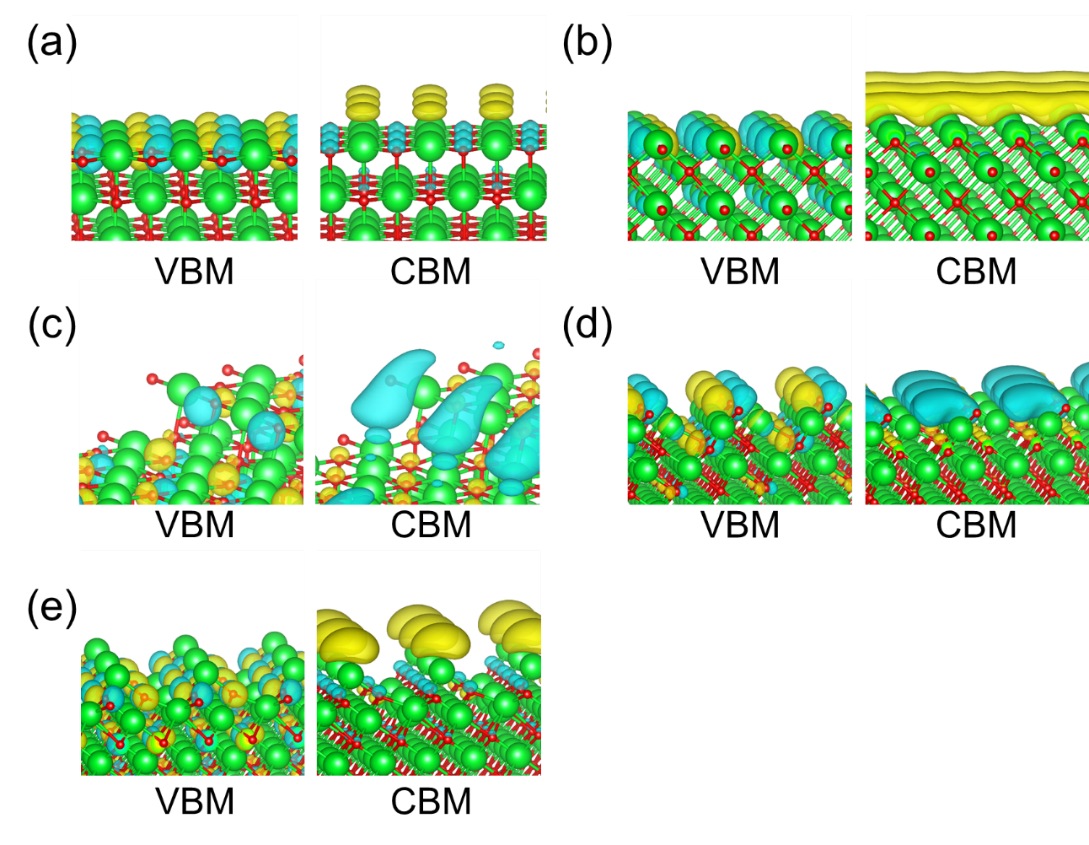


BaO


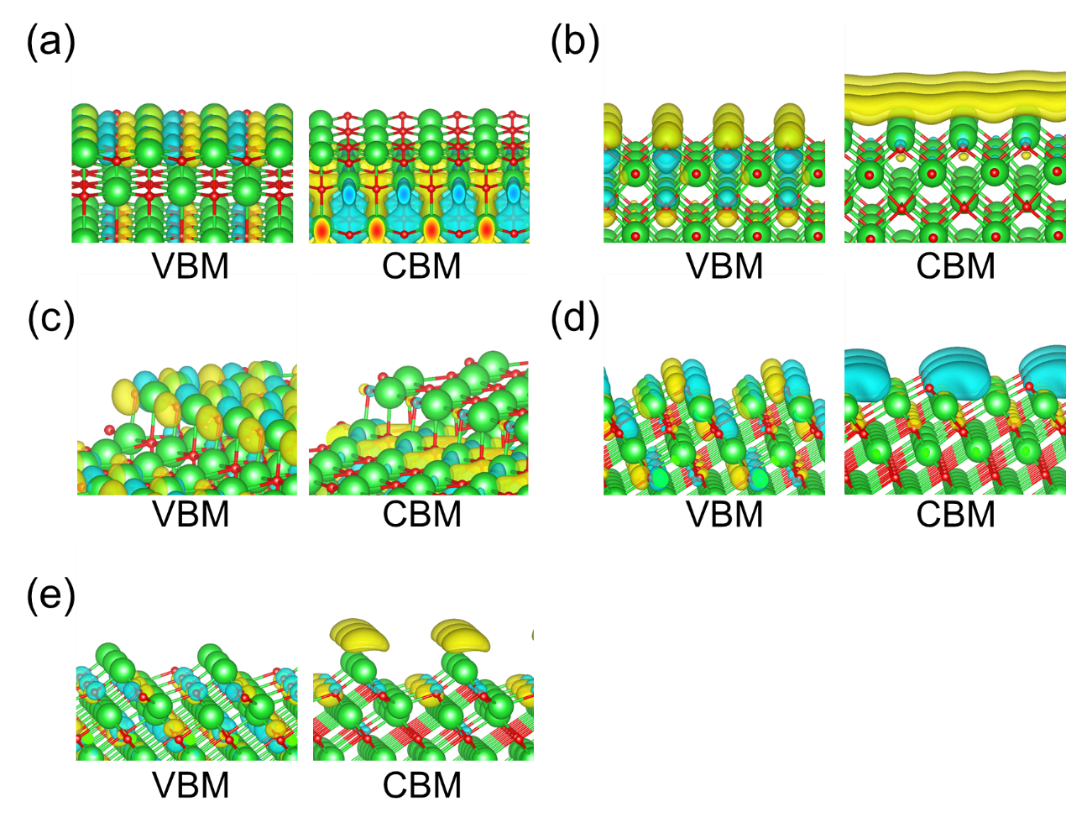


**Figure S3.** Crystal orbitals of VBM and CBM for MgO, CaO, SrO and BaO surfaces: (a) (100) surface, (b) (110) surface, (c) stepped (100) surface, (d) O-oct surface and (e) M-oct surface. The red sphere represents an O atom. The other colors indicate alkaline-metal atoms.


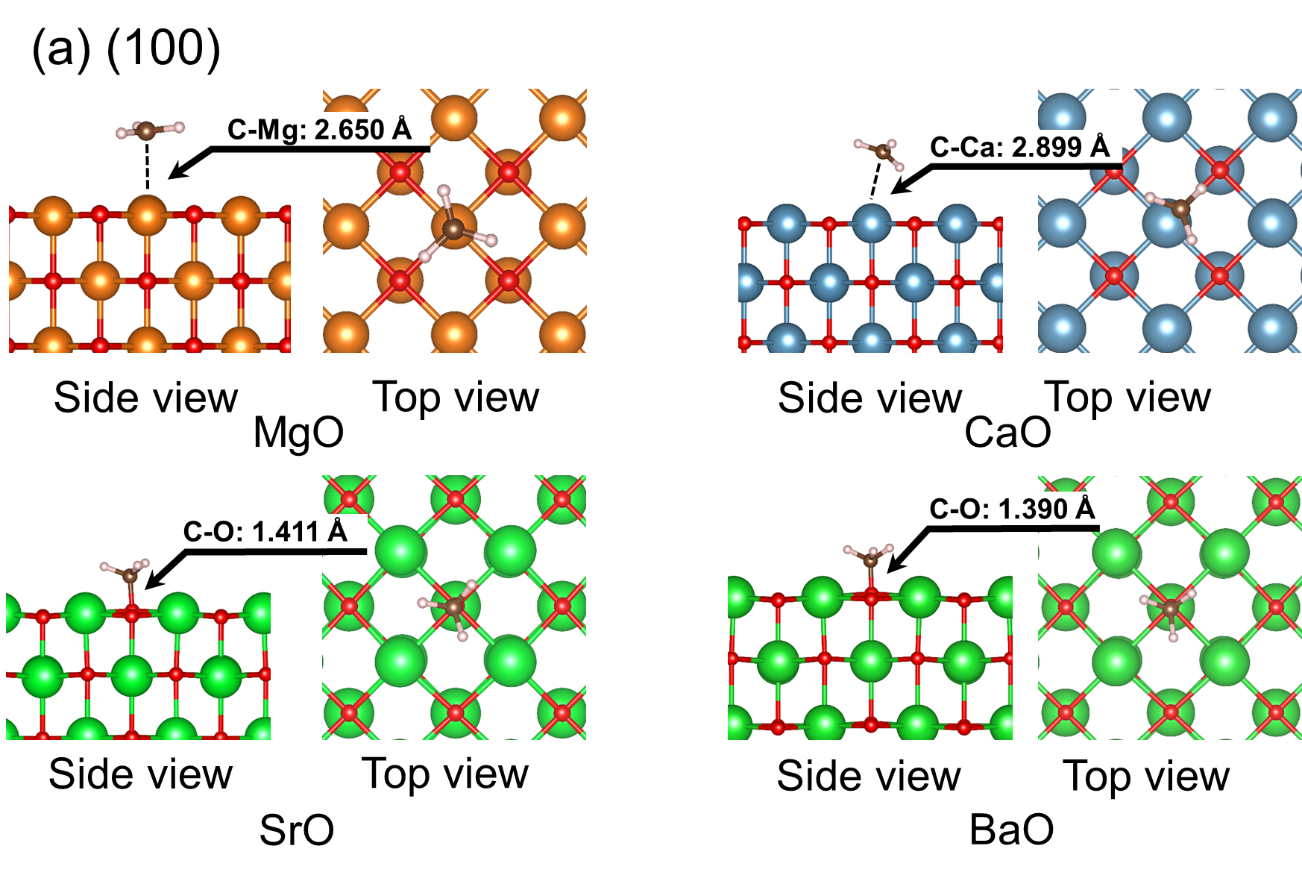


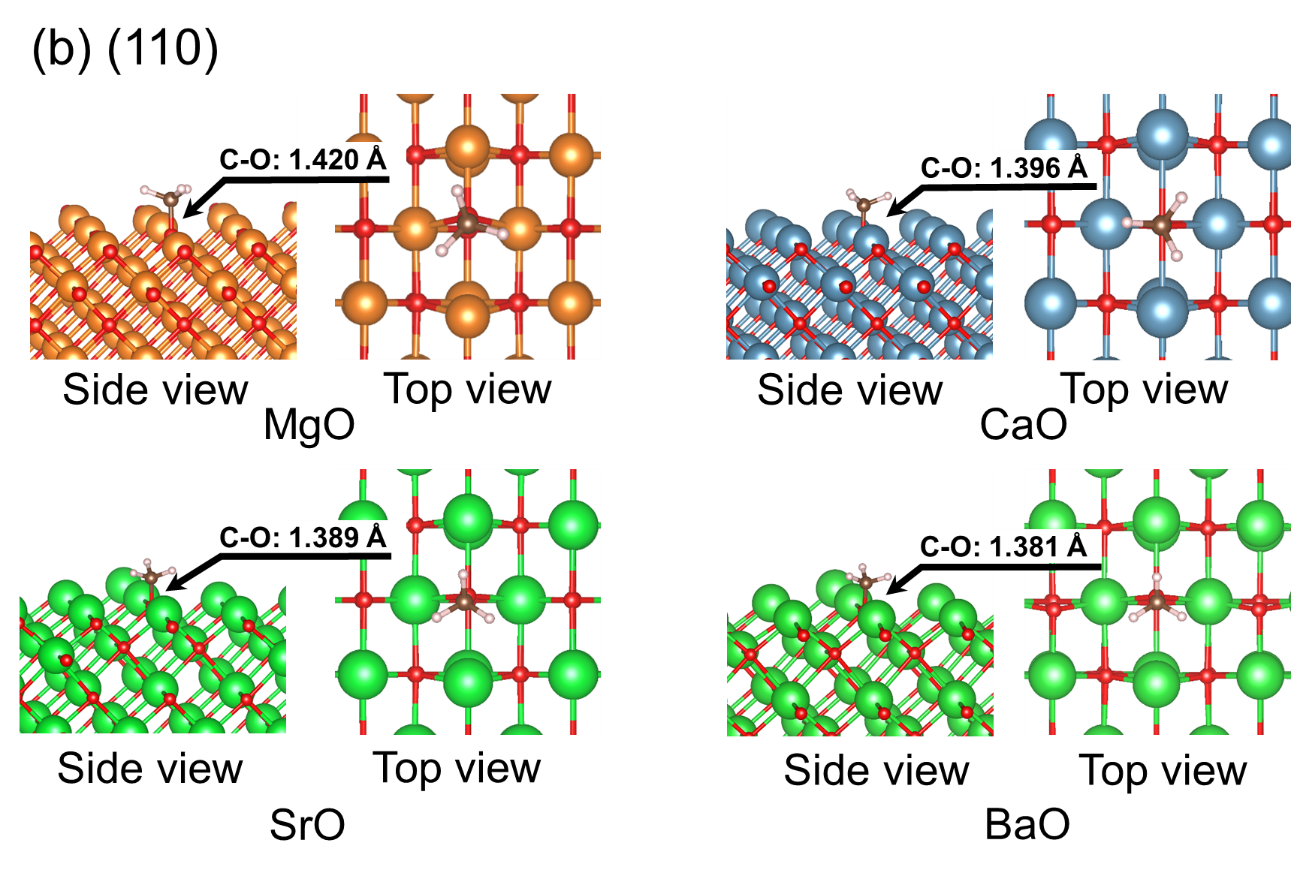


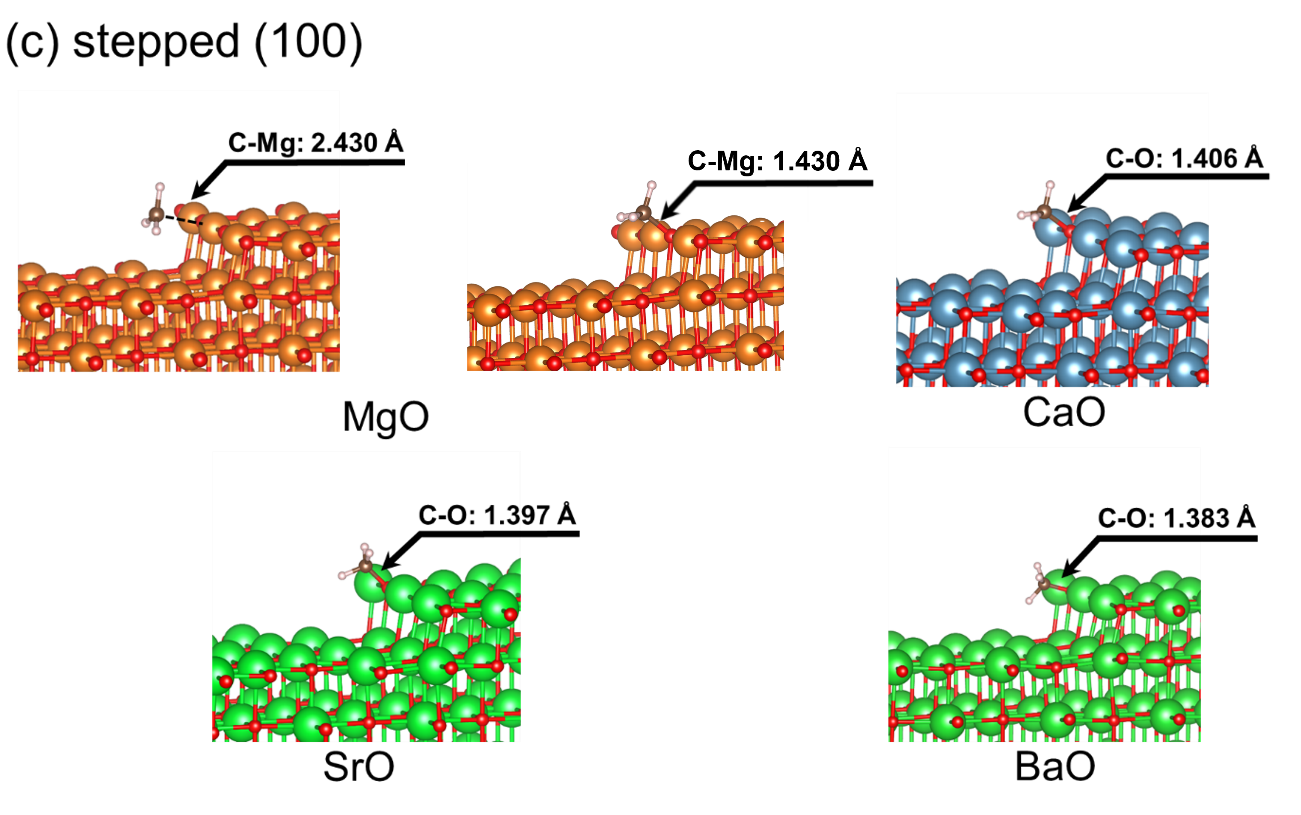


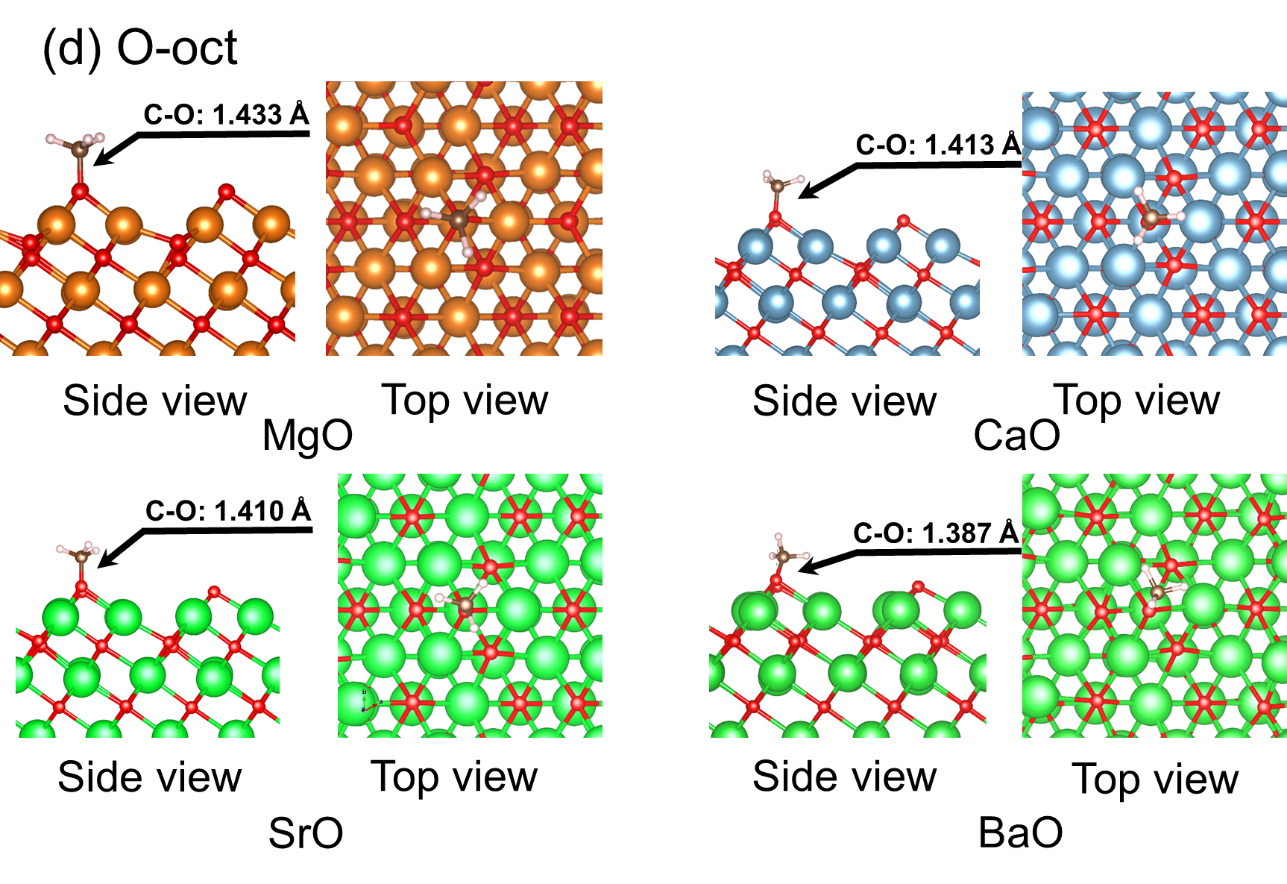


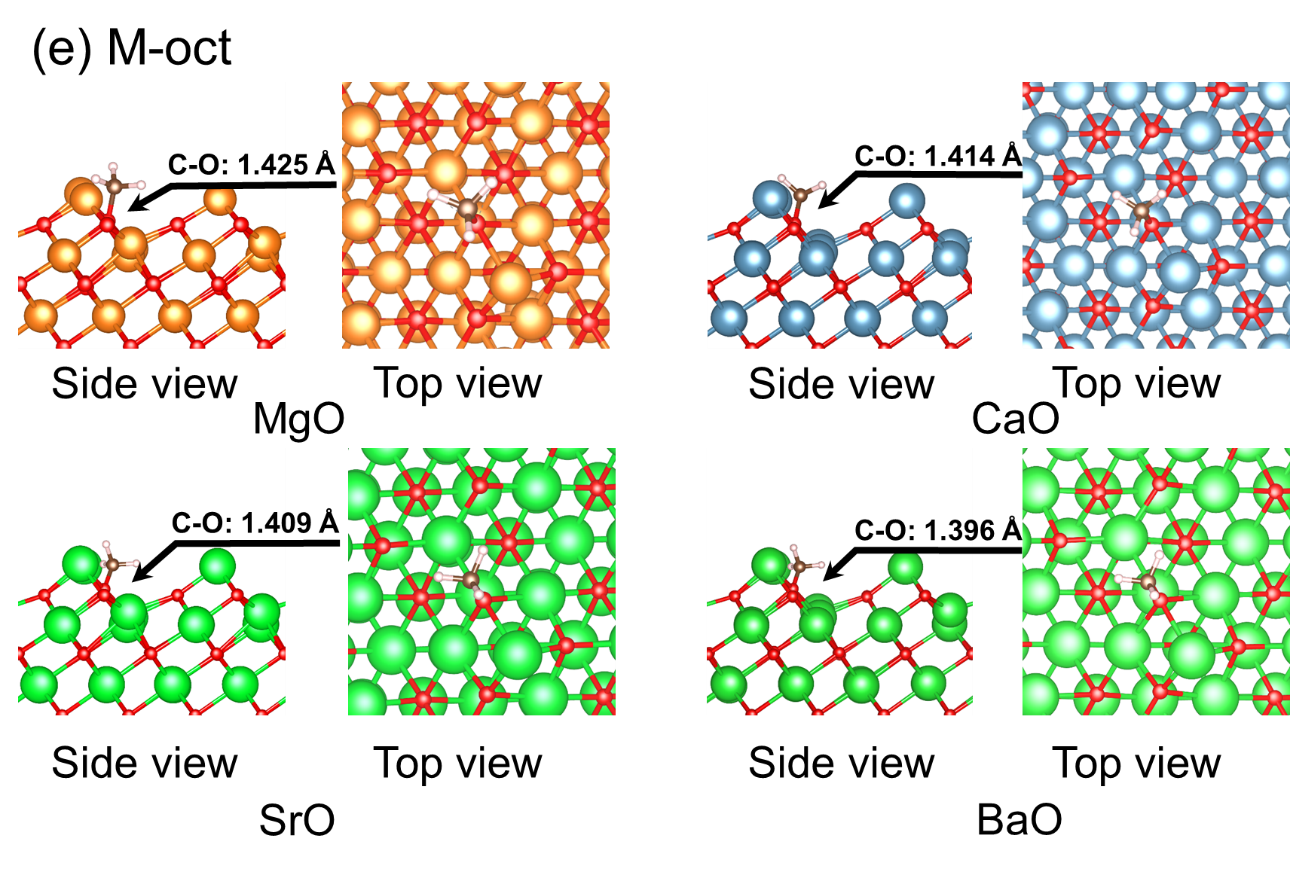


**Figure S4.** Adsorption structures for the methyl radical on the MgO, CaO, SrO and BaO, surfaces: (a) (100) surface, (b) (110) surface, (c) stepped (100) surface, (d) O-oct surface and (e) M-oct surface. The white, brown, and red spheres represent H, C, and O atom, respectively. The other colors indicate alkaline-metal atoms.


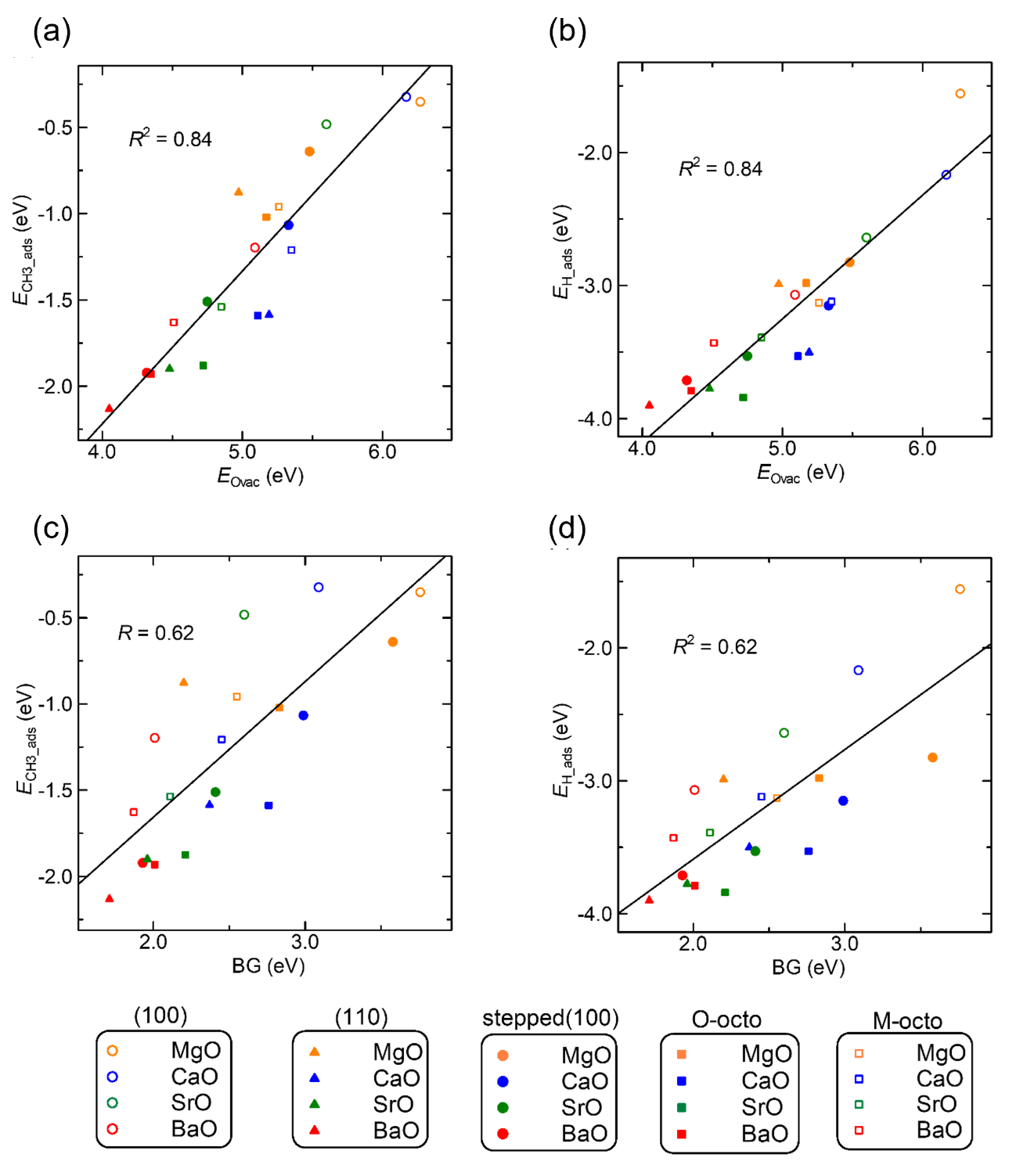


**Figure S5.** Correlation between (a) between *E*_Ovac_ and *E*_CH3_ads_, (b) between *E*_Ovac_ and *E*_H_ads_, (c) between BG and *E*_CH3_ads_, and (d) between BG and *E*_H_ads_.


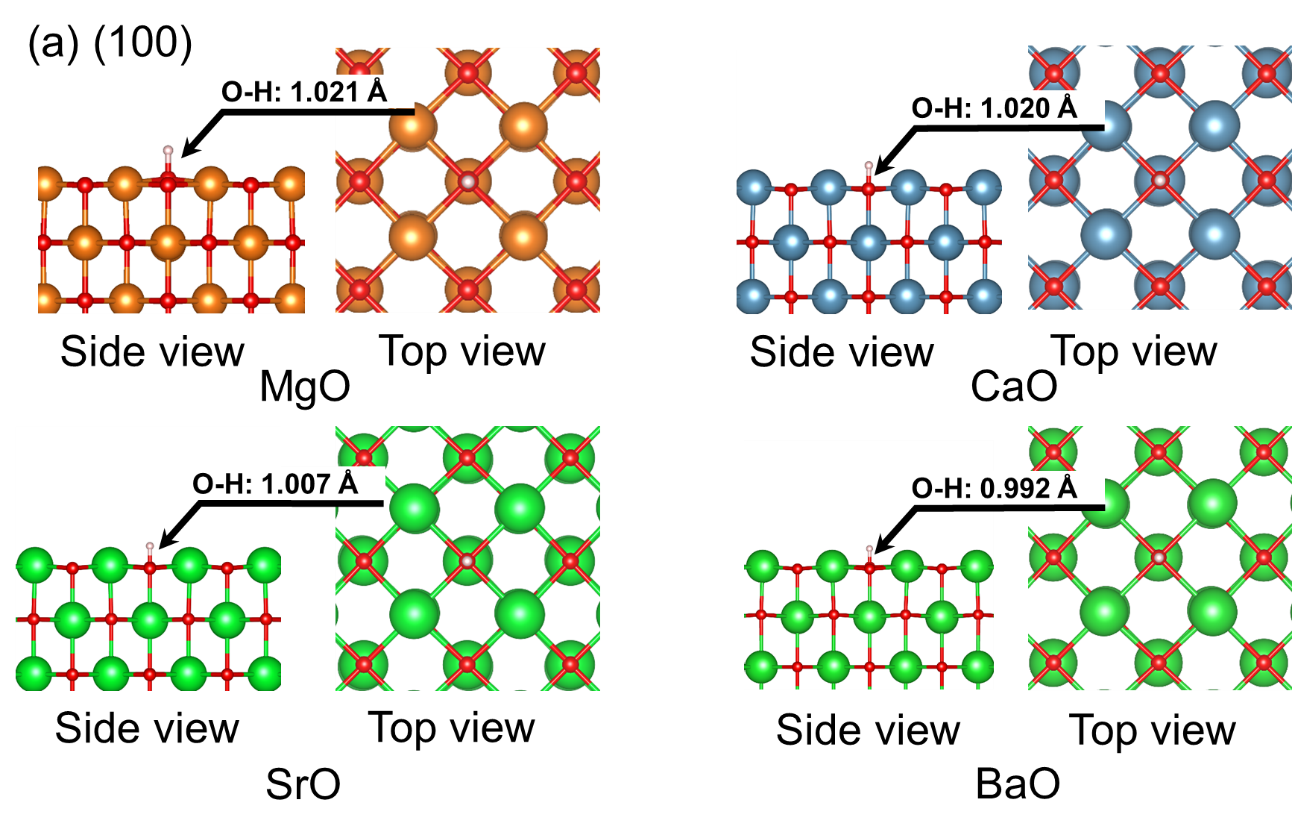


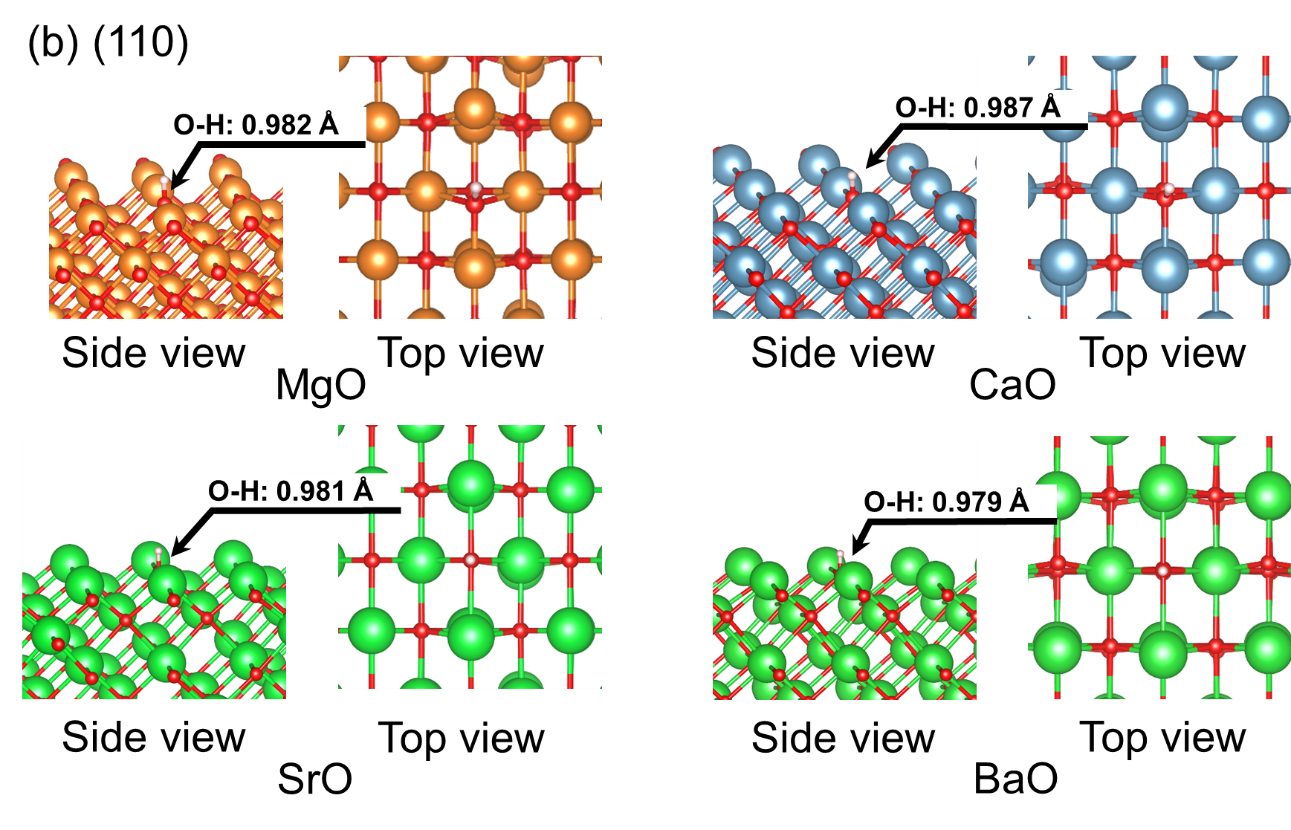


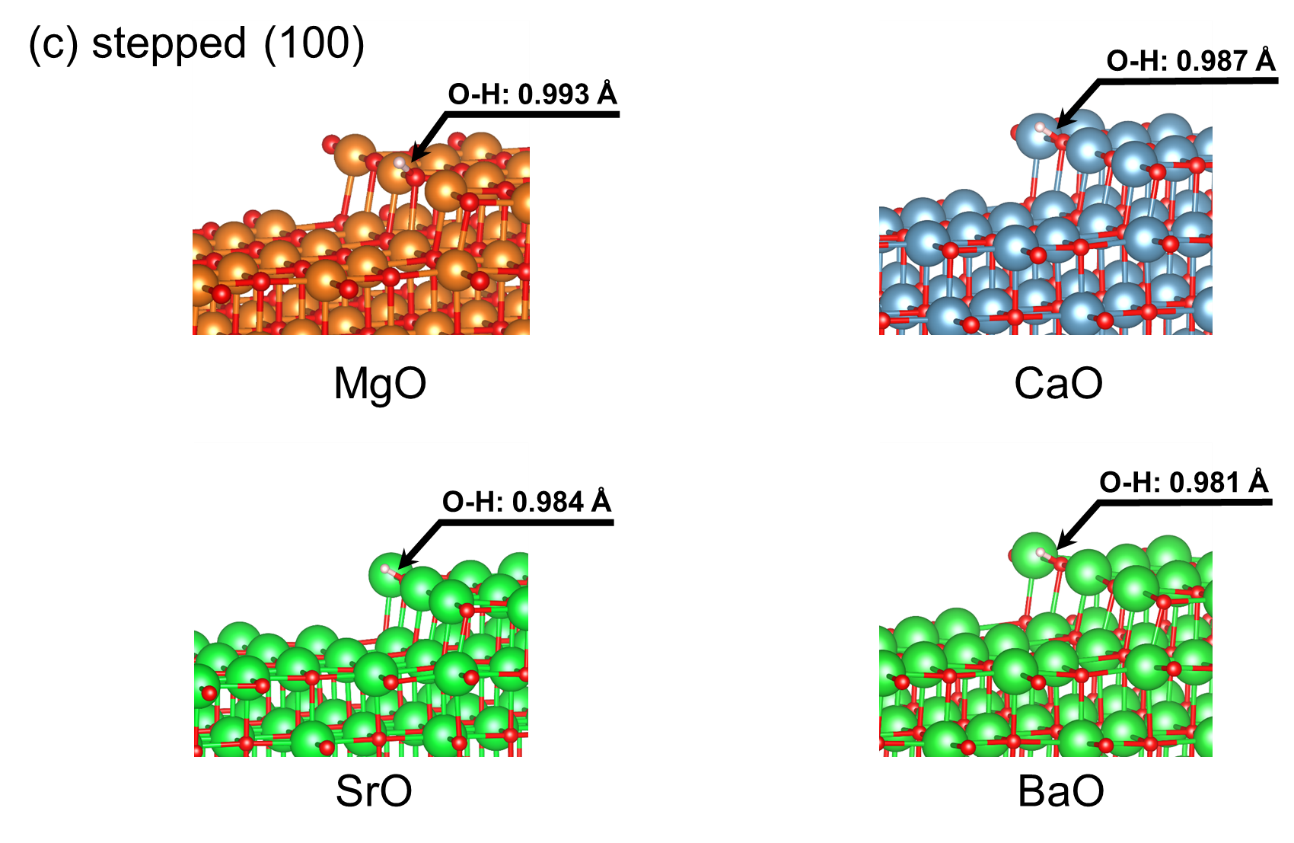


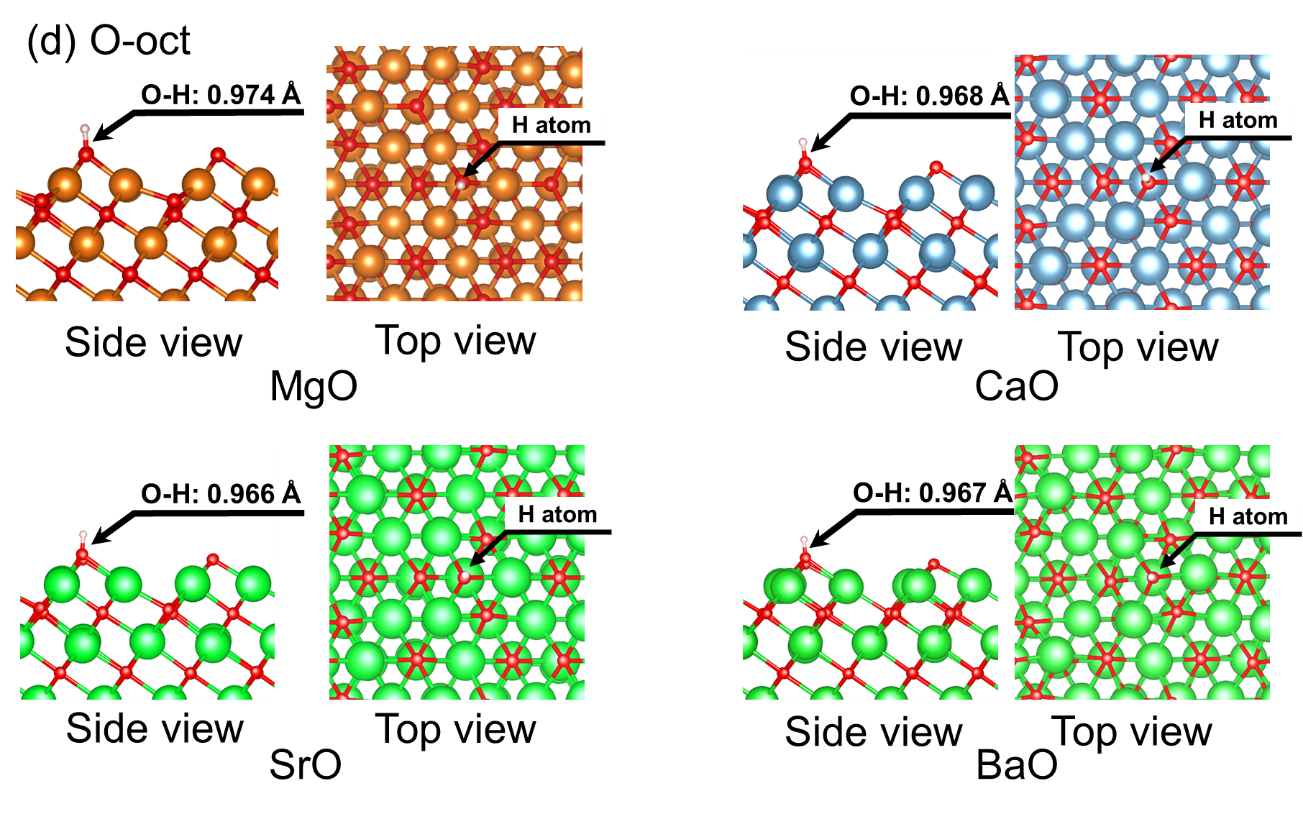


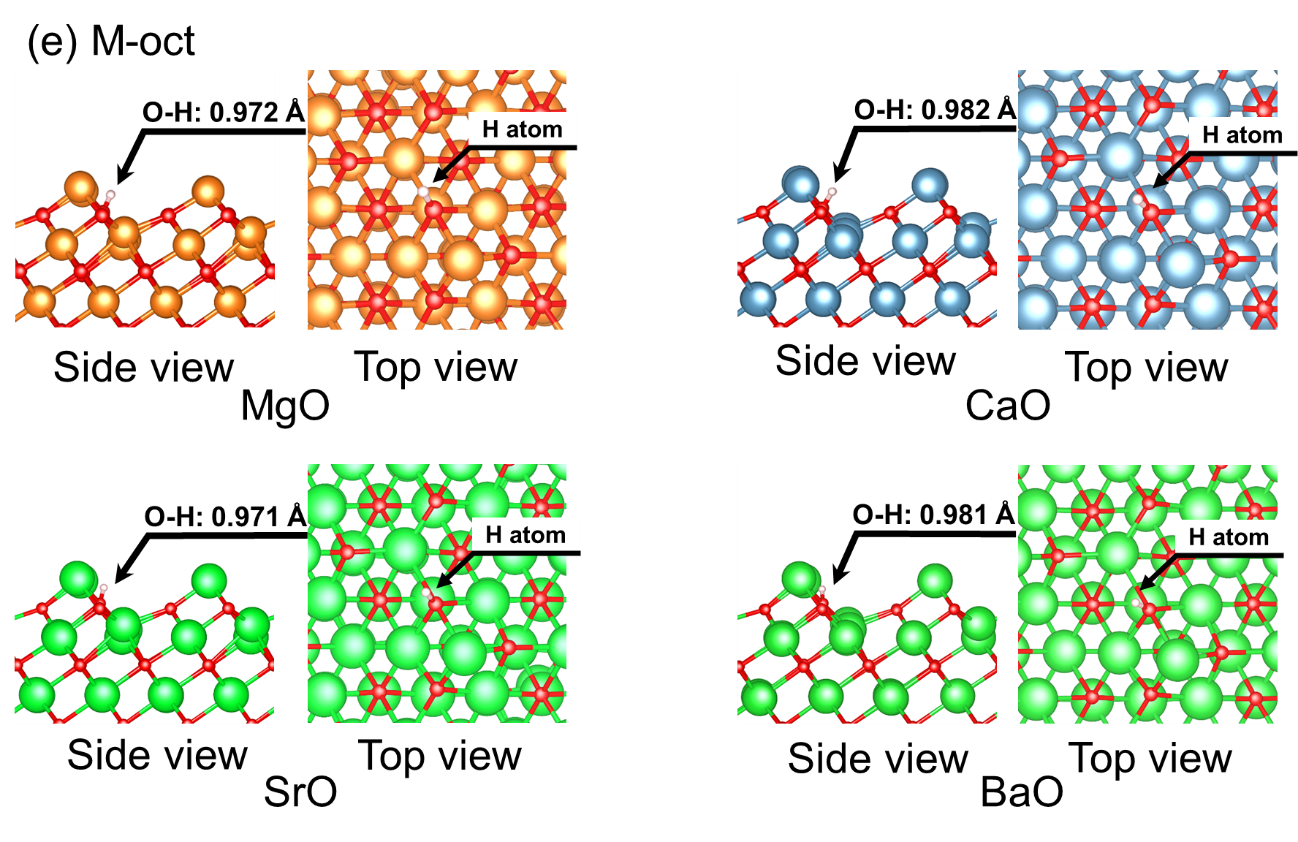


**Figure S6.** Adsorption structures for hydrogen atom on the MgO, CaO, SrO and BaO, surfaces: (a) (100) surface, (b) (110) surface, (c) stepped (100) surface, (d) O- oct surface and (e) M- oct surface. The white and red spheres represent H and O atoms. The other colors indicate alkaline-metal atoms.


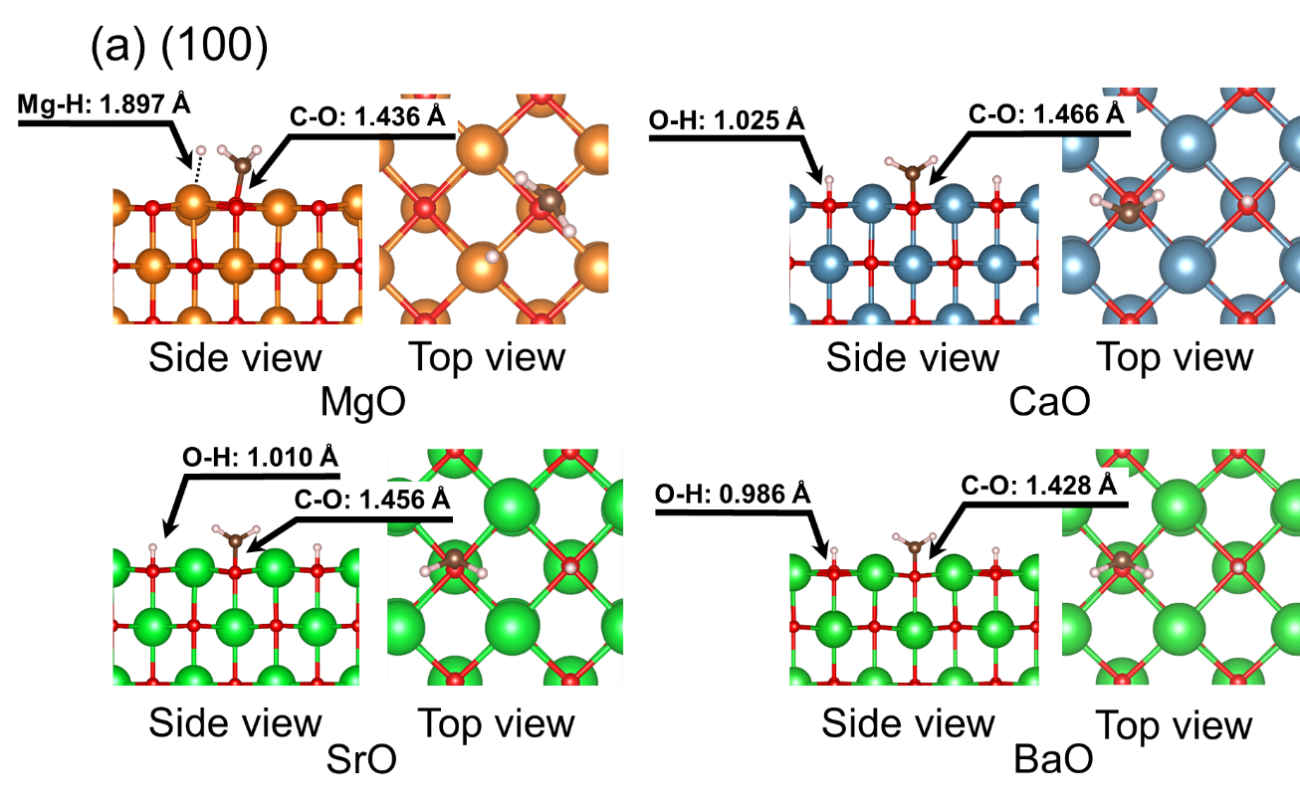


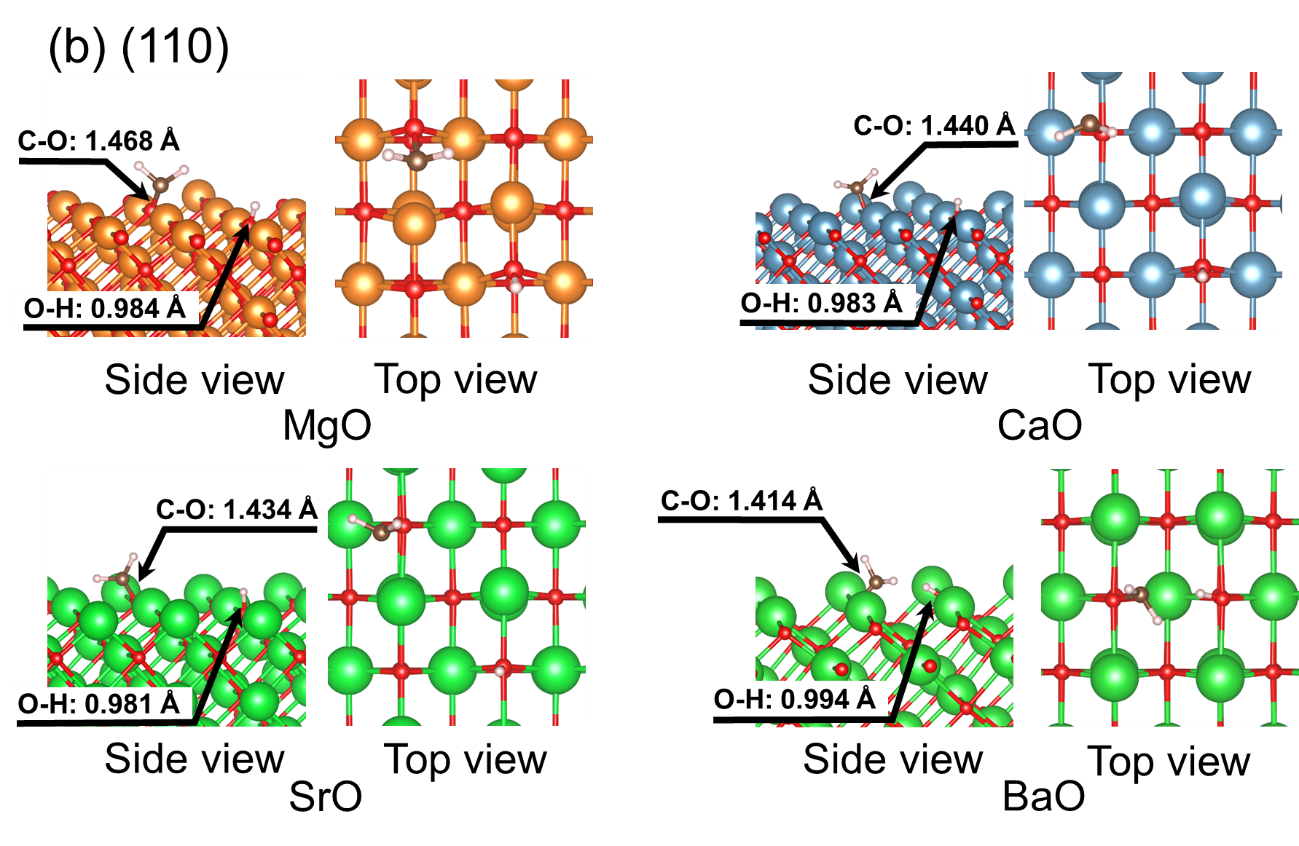


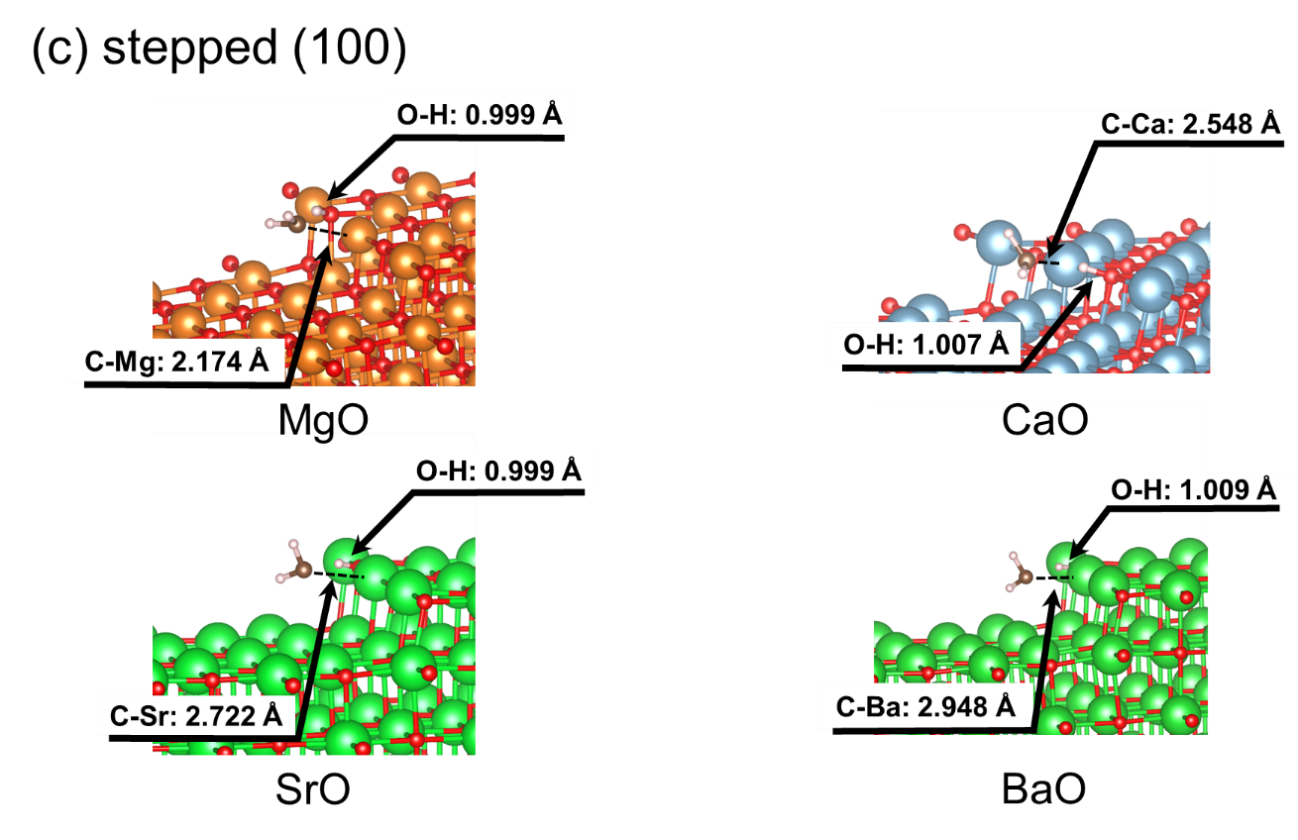


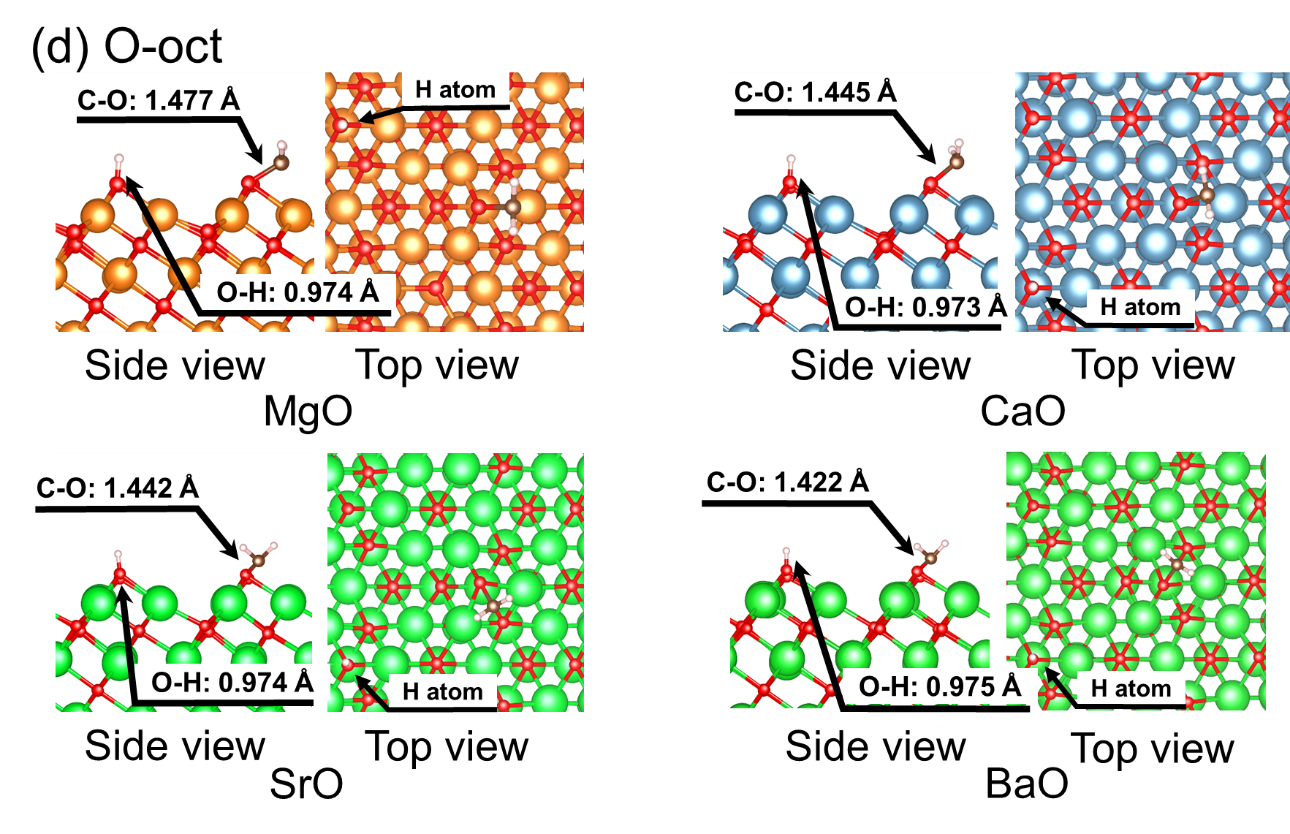


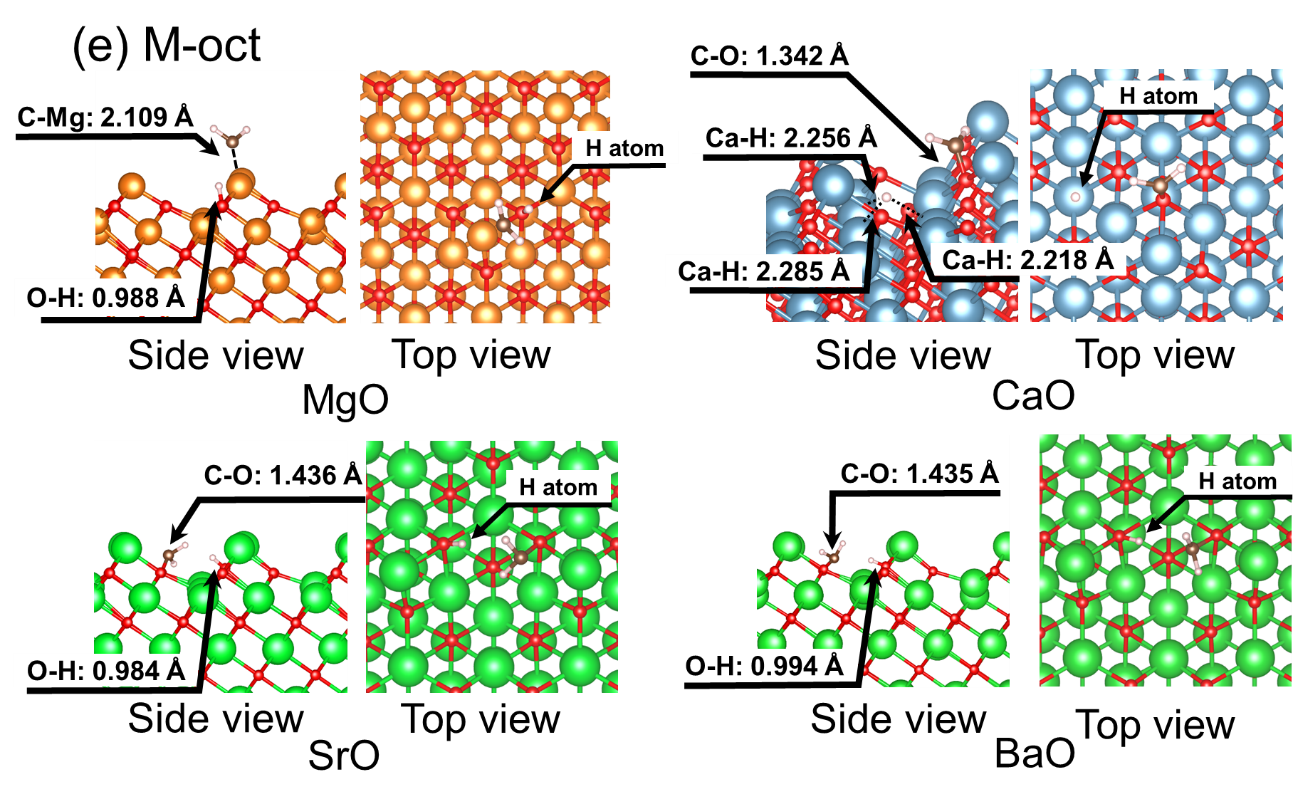


**Figure S7.** Adsorption structures for methylene and hydrogen atom on the MgO, CaO, SrO, BaO, surfaces: (a) (100) surface, (b) (110) surface, (c) stepped (100) surface, (d) O-oct surface and (e) M-oct surface. The white, brown, and red spheres represent H, C, and O atoms, respectively. The other colors indicate alkaline-metal atoms.


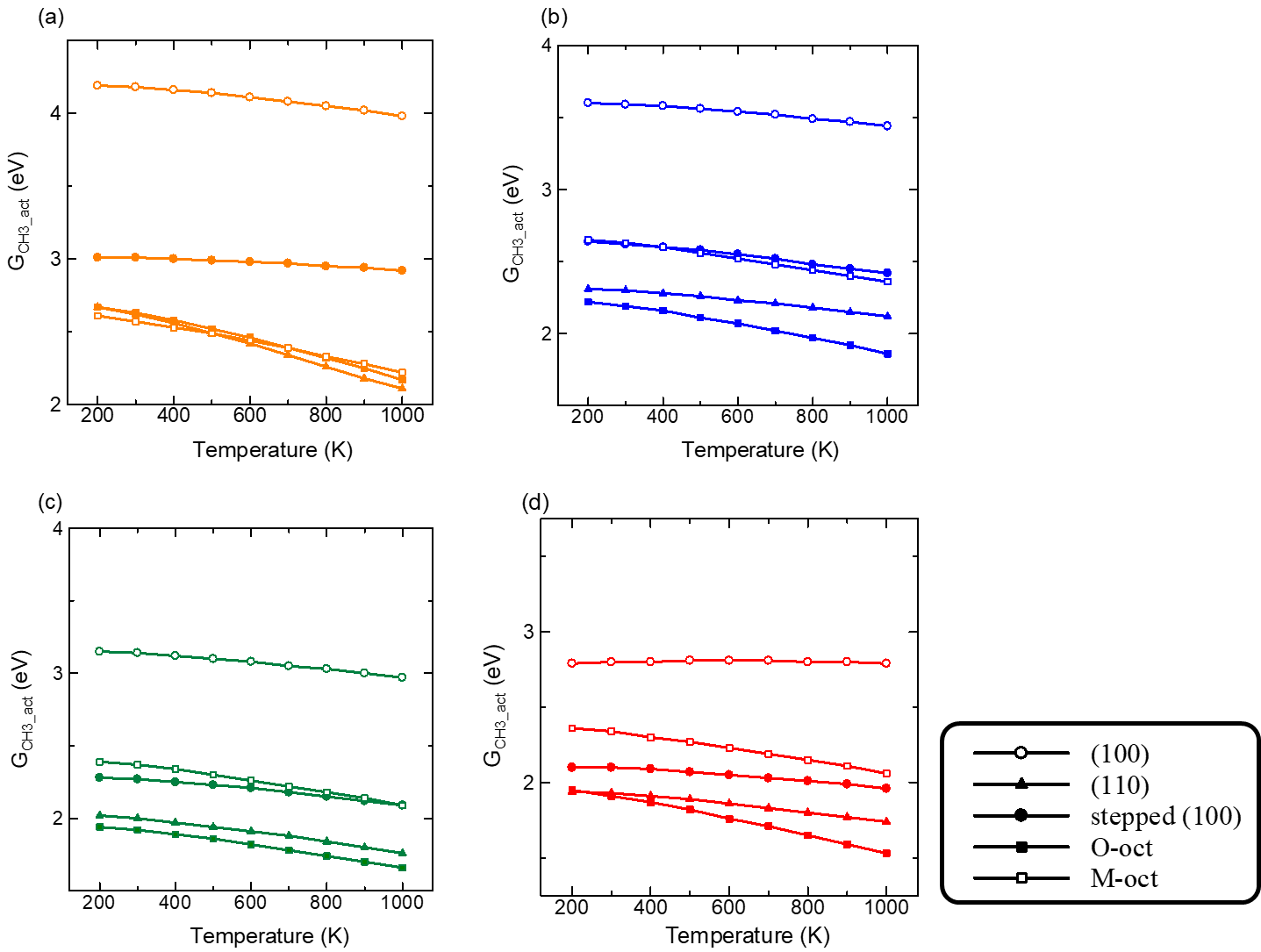


**Figure S8.** *G*_CH3_act_ values with the change in temperature: (a) MgO, (b) CaO, (c) SrO and (d) BaO.
